# Mechanism of Oxygen Quenching of the Excited States of Heteroleptic Chromium(III) Phenanthroline Derivatives

**DOI:** 10.1021/acs.inorgchem.3c02343

**Published:** 2023-09-18

**Authors:** Ahmed M. M. Alazaly, Guy J. Clarkson, Michael D. Ward, Ayman A. Abdel-Shafi

**Affiliations:** † Department of Chemistry, Faculty of Science, Ain Shams University, Abbassia, Cairo 11566, Egypt; ‡ Department of Chemistry, University of Warwick, Coventry CV4 7AL, U.K.

## Abstract

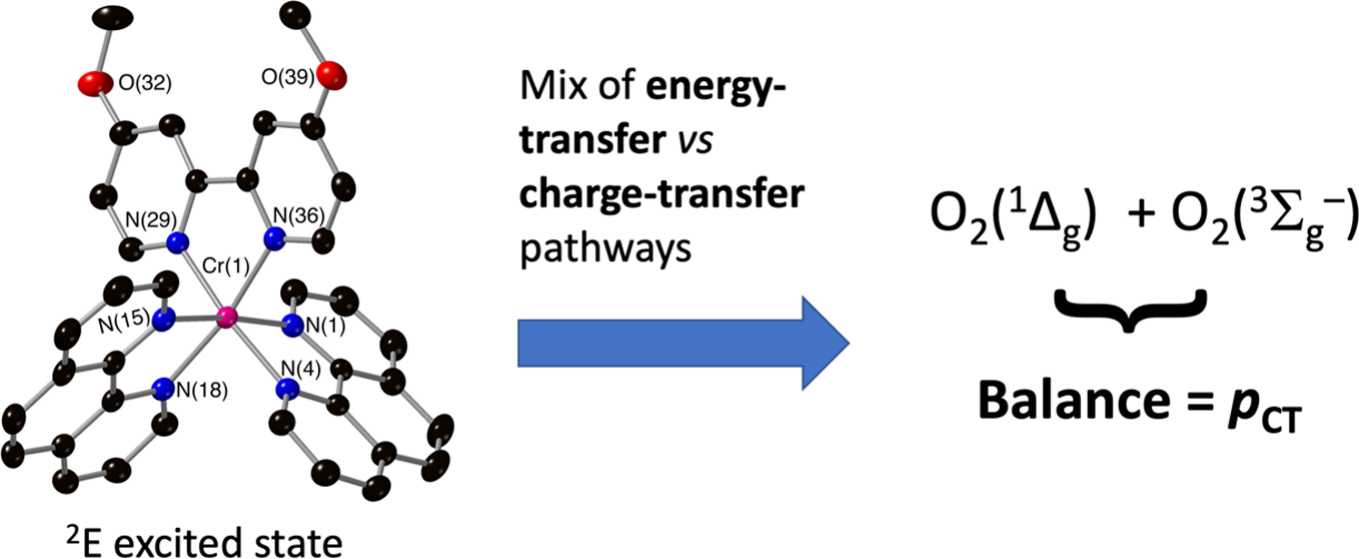

In this study, we report the synthesis and characterization of some heteroleptic Cr(III) complexes of the form [Cr(Phen)_2_L](OTf)_3_, where Phen = 1,10-phenanthroline and L is either 2,2′-bipyridine (bpy) or its derivatives, such as 4,4′-dimethyl-2,2′-bipyridine (4,4′-DMB), 4,4′-dimethoxy-2,2′-bipyridine (4,4′-DMOB), 4,4′-di*tert*-butyl-2,2′-bipyridine (4,4′-d^t^bpy), 5,5′-dimethyl-2,2′-bipyridine (5,5′-DMB), 4,4′-dimethoxycarbonyl-2,2′-bipyridine (4,4′-dmcbpy) or 1,10-phenanthroline derivatives, such as 5-methyl-1,10-phenanthroline (5-Me-Phen) and 4,7-dimethyl-1,10-phenanthroline (4,7-DMP). Heteroleptic complexes were prepared in two stages *via* the intermediate [Cr(Phen)_2_(CF_3_SO_3_)_2_](CF_3_SO_3_) and five examples have been crystallographically characterized. Steady-state absorption and luminescence emission characteristics of these complexes were measured in 1 M HCl solutions. The luminescence quantum yield of these complexes was found to be the lowest for [Cr(Phen)_2_(4,4′-dmcbpy)](OTf)_3_ and the highest for [Cr(Phen)_2_(4,4′-DMB)](OTf)_3_ with values of 0.31 × 10^–2^ and 1.48 × 10^–2^, respectively. The calculated excited state energy, E_0–0_, was found to vary within the narrow range of 163.1–165.0 kJ mol^–1^ across the series. Transient absorption spectra in degassed, air-equilibrated, and oxygen-saturated 1 M HCl aqueous solutions were also measured at different time decays and demonstrated no significant differences, indicating the absence of any ion-separated species in the excited state. Excited-state decay traces at the wavelength of maximum absorption were used to calculate oxygen quenching rate constants, *k*_q_, which were found to be in the range 3.26–5.27 × 10^7^ M^–1^ s^–1^. Singlet oxygen luminescence photosensitized by these complexes was observed in D_2_O, and its luminescence intensity at 1270 nm was used for the determination of singlet oxygen quantum yields for these complexes, which were in the range of 0.20–0.44, while the fraction of the excited ^2^E state quenched by oxygen was in the range of 0.22–0.68, and the efficiency of singlet oxygen production was in the range of 0.44–0.90. The mechanism by which the excited ^2^E state is quenched by oxygen is explained by a spin statistical model that predicts the balance between charge transfer and noncharge transfer deactivation pathways, which was represented by the parameter *p*_CT_ that was found to vary from 0.35 to 0.68 for this series of Cr(III) complexes.

## Abstract 2

The balance of energy-transfer and electron-transfer mechanisms involved in quenching of excited states of Cr(III) tris-diimine complexes is explained by a spin statistical model that predicts the balance between charge transfer and noncharge transfer deactivation pathways, represented by the parameter *p*_CT_, without requiring electrochemical data.

## Introduction

The photophysical properties of chromium(III) polypyridyl complexes make them appealing for a wide range of potential applications due to the long-lived excited states of hundreds of microseconds and their thermal stability. They are excellent materials for solar energy conversion,^1−3^ the photodegradation of pollutants in the environment via singlet oxygen generation,^4−7^ as luminescent probes for the detection of hydrophilic and hydrophobic sites in polymeric solutions and films,^8^ for the photo-oxidation of phenol and the electroreduction of NO in self-assembled electrodes,^9,10^ and as potential agents for DNA photocleavage.^11,12^ It has been reported that the luminescence lifetime and excited-state absorption decay measurements depend strongly on the structure of the ligands, the presence of added salts, and pH conditions.^13^ [Cr(Phen)_3_]^3+^ and [Cr(Phen)_2_(dppz)]^3+^ (dppz = dipyridophenazine) were reported to bind strongly to the protein apoTf, leading to promising applications in photodynamic therapy, as this protein could act as a good carrier for these complexes.^14^

The electronic properties of the Cr(III) center can be tuned by the judicious choice of the ancillary dipyridyl-type ligands (NN). The synthesis of heteroleptic Cr(III) dipyridyl complexes is not straightforward, as efforts to activate the inert metal center often result in ligand scrambling.^15^ Nevertheless, a recently reported methodology employing [(NN)_2_Cr(OTf)_2_]^+^ complexes as synthons^15−17^ shows the way to a new class of molecular species with the potential for efficient hole injection into semiconductor substrates. Herein, we describe the preparations of, as well as photophysical investigations into, a family of structurally related homoleptic and heteroleptic Cr(III) dipyridyl complexes, focusing in particular on the quenching of their excited state by O_2_.

Molecular oxygen is one of the most known effective quenchers of electronically excited states, as it has two low-lying electronically excited states, O_2_(^1^Δ_g_) and O_2_(^1^Σ_g_^+^), with energies of 94 and 157 kJ mol^–1^, respectively, above the ground state, O_2_(^3^Σ_g_^–^).^18,19^ The first detailed mechanism of oxygen quenching of excited triplet states was proposed by Gijzeman *et al.* based on spin statistical factor quenching pathways.^20^ This mechanism was modified by Garner and Wilkinson by including charge transfer intermediates and intersystem crossing between them,^21^ and later by Wilkinson and Abdel-Shafi, who suggested direct energy transfer via the singlet channel that leads to the direct formation of O_2_(^1^Δ_g_), in addition to O_2_(^1^Δ_g_) formation through the charge transfer intermediate through a singlet channel.^22,23^ Energy dissipation through the triplet pathway leads to the formation of the ground state of both the sensitizer and oxygen. Schmidt *et al.* have developed a model that describes the balance between noncharge transfer and charge transfer deactivation channels by the parameter *p*_CT_.^24−30^ Singlet oxygen generated by the mechanisms described above is a potent oxidant, playing a key role in fine chemical synthesis, photosensitized oxidations, photodynamic therapy, photodynamic inactivation of viruses and cells, photosterilization of blood components, photodegradation of dyes and polymers, and water-borne bacteria inactivation.^31−39^

Studies on singlet oxygen photosensitization by coordination compounds are very limited in comparison with those reported for aromatic compounds.^18,19,40,41^ The majority of these studies have been concerned on the study of singlet oxygen generation photosensitized by ruthenium(II) polypyridyl complexes in aqueous and nonaqueous media.^41−56^ Photosensitization of singlet oxygen by metal complexes other than Ru(II) has also been reported^57−69^ using, for example, complexes of Pd(II) and Pt(II),^57−61,65−68^ Os(II) with terpyridyl ligands,^64^ Au(I),^62^ Ir(III),^63,65,66^ and Re(I).^69^

Interest in oxygen quenching of excited-state Cr(III) complexes was started with the work of Pfeil more than half a century ago.^70^ He reported a very wide range of oxygen quenching rate constants from ≤10^7^ to 10^11^ M^–1^ s^–1^ in fluid solution. He classified the complexes into two groups according to the observed oxygen quenching rate constants: these were (i) complexes with insulator ligands, such as ethylenediamine and trismethylenediamine, displaying relatively small quenching rate constants (≤10^7^ M^–1^ s^–1^), and (ii) complexes with conducting ligands that have extensive π-electron systems, such as cyanide, acetylacetone, and isothiocyanate, which displayed larger quenching rate constants in the range 10^8^ to 10^11^ M^–1^ s^–1^. Brunschwig and Sutin also studied oxygen quenching rate constants of [Cr(diimine)_3_]^3+^ complexes and tried to correlate the quenching rate constants with the experimentally achievable reduction potential of the complexes and found none.^71^ In contrast, Serpone *et al.* correlated oxygen quenching rate constants for a series of homoleptic Cr(III) complexes with bipyridine and phenanthroline ligands and their derivatives, with the free energy change of energy transfer, and discussed the correlation based on the Marcus inverted region despite the approximately constant value of the free energy change of energy transfer.^1^ The first singlet oxygen detection, from the quenching of [Cr(1,4,7-triazacyclononane)(NCS)_3_], was demonstrated chemically by Kirk *et al.*, who reported a value of 0.48 for the ^1^O_2_ formation quantum yield.^72^ The first spectroscopic detection of ^1^O_2_ was obtained by Tiyabhorn and Zahir, who found a quantum yield of 0.86 for the quenching by ^3^O_2_ of the excited doublet state of [Cr(bpy)_3_]^3+^ in D_2_O.^73^ Otto *et al.* have recently qualitatively explained the quenching efficiency of the excited state of [Cr(ddpd)_2_]^3+^ (where ddpd = *N,N*′-dimethyl-*N,N*′-dipyridine-2-yl-pyridine-2,6-diamine) by molecular oxygen in water on the basis of (i) the very long excited state lifetime of this complex and (ii) spin statistics and the quenching rate constant values associated with an effective shielding of Cr(III) by the ligands and the counterions.^74^ Recently, Wenger and co-workers have studied the oxidizing properties of spin-flipped excited states in a [Cr(dpq)_2_]^3+^ (dpq = 2,6-bis(8′-quinolinyl)pyridine) in comparison with [Ru(bpz)_3_]^2+^ (bpz = 2,2-bipyrazine) and found that the similarity of the driving force dependence for photoinduced electron transfer from 10 different aromatic donors to the excited [Cr(dqp)_2_]^3+^ and [Ru(bpz)_3_]^2+^ establishes that the excited-state oxidation potential of [Cr(dqp)_2_]^3+^ approaches that of [Ru(bpz)_3_]^2+^.^10^

Heinze and co-workers have recently studied long-lived phosphorescence from spin-flipped Cr(II) complexes with bulky substituents attached to a tridentate ligand in order to study the shielding effect of these bulky substituents in shielding the metal center from oxygen, which was found to be more effective in metal-centered emitters than charge transfer analogues.^75^

Long-lived luminescence from first-row transition metal complexes is very challenging to achieve and therefore of high interest;^76^ despite this, the number of publications on luminescent Cr(III) complexes and their quenching by oxygen is limited. The spin difference between the ground quartet state and the excited doublet state of Cr(III) complexes imposes another challenging target for studying the mechanism of oxygen quenching to these excited states and adds the spin-state change on deactivation to the mechanism of quenching as an extra factor affecting the mechanism of quenching. In addition, the metal-centered nature of the excited state of Cr(III) complexes is another interesting factor in the quenching mechanism in comparison with the metal-to-ligand charge transfer nature of Ru(II) and other d^6^ metal complexes.

In this study, the synthesis and characterization of a series of heteroleptic Cr(III) phenanthroline derivatives are reported. Steady-state absorption and luminescence emission spectra, as well as the emission lifetime of the studied complexes, are presented. Oxygen quenching of the excited ^2^E states of these complexes is reported, and ^1^O_2_ formation quantum yields following photosensitization by these complexes in D_2_O are also presented. The mechanism of quenching by ^3^O_2_, and the resultant production of ^1^O_2_, is studied in detail for Cr(III) complexes, showing the balance between charge transfer and noncharge transfer quenching pathways quantitatively using a kinetic model previously applied for oxygen quenching of the excited triplet state of aromatic hydrocarbons and successfully for oxygen quenching of excited states with a charge transfer nature. Despite the experimental difficulties of measuring the oxidation potential of these metal complexes, we were able to quantitatively assign the fraction of the charge transfer contribution to the quenching mechanism.

## Experimental Section

The synthesis of homoleptic and heteroleptic tris-diimine Cr(III) complexes was performed in air with atmospheric moisture excluded by the use of an anhydrous CaCl_2_-filled drying tube. The commercially obtained ligands of the highest available purity were obtained from the following sources: 1,10-phenanthroline (Phen), 2,2′-bipyridine (bpy), 4,4′-dimethyl-2,2′-bipyridine (4,4′-DMB), 4,4′-dimethoxy-2,2′-bipyridine (4,4′-DMOB), and 2,2′-bipyridine-4,4′-dicarboxylic acid (4,4′-dcbpy) from Sigma-Aldrich; 4,4′-di*tert*-butyl-2,2′-bipyridine (4,4′-d^t^bpy) from Fluorochem; 5,5′-dimethyl-2,2′-bipyridine (5,5′-DMB), 5-methyl-1,10-phenanthroline (5-Me-Phen), and 4,7-dimethyl-1,10-phenanthroline (4,7-DMP) from Alfa Aesar; trifluoromethanesulfonic acid (99%) from Acros organics. Dichloromethane (DCM), ethanol (EtOH), acetonitrile (ACN), and deuterium oxide (D_2_O, 99.9% D) were purchased from Sigma-Aldrich. 4,4′-Dimethoxycarbonyl-2,2′-bipyridine-(4,4′-dmcbpy) was synthesized from 2,2′-bipyridine-4,4′-dicarboxylic acid as reported in the literature.^77^ Concentrated (37%) hydrochloric acid (HCl) was purchased from Alfa Aesar. All reagents were used as received without further purification.

### Synthesis of Precursor Complexes

The complex *cis-*[Cr(Phen)_2_Cl_2_]Cl was synthesized as reported in the literature using either methanol or ethanol as the solvent;^78^ this was converted to *cis-*[Cr(Phen)_2_(CF_3_SO_3_)_2_](CF_3_SO_3_) in essentially quantitative yield by reaction with triflic acid for 24 h under N_2_.^79^ From this, synthesis of heteroleptic complexes as reported below follows literature methodologies.^15,80,81^

#### Synthesis of Homoleptic [Cr(Phen)_3_](OTf)_3_ (**1**)

[Cr(Phen)_2_(CF_3_SO_3_)_2_](CF_3_SO_3_) (1 g, 1.16 mmol) and Phen (524 mg, 2.91 mmol) were suspended in CH_2_Cl_2_ (80 mL). The reaction mixture was heated to reflux for 26 h, and a yellow precipitate was formed. This was separated by filtration, washed with CH_2_Cl_2_ (2 × 30 mL), and dried in vacuo. [Cr(Phen)_3_](OTf)_3_ was isolated as a yellow powder (0.92 g, 76%). IR: ν_C=N_ 1607 cm^–1^. ES^+^MS (CH_3_CN): *m*/*z* 890.0 ([**1** – OTf]^+^), 197.4 ([**1** – 3OTf]^3+^). Anal. Calcd for C_39_H_24_N_6_CrF_9_O_9_S_3_: C, 45.05; H, 2.33; N, 8.08. Found: C, 44.99; H, 2.17; N, 8.01%.

#### Synthesis of [Cr(Phen)_2_(bpy)](OTf)_3_ (**2**)

[Cr(Phen)_2_(CF_3_SO_3_)_2_](CF_3_SO_3_) (1 g, 1.16 mmol) and bpy (527 mg, 3.37 mmol) were suspended in CH_2_Cl_2_ (90 mL). The reaction mixture was heated to reflux for 30 h, and a yellow precipitate was formed. This was separated by filtration, washed with CH_2_Cl_2_ (2 × 20 mL), and dried in vacuo. [Cr(Phen)_2_(bpy)](OTf)_3_ was isolated as a yellow powder (1.06 g, 90%). IR: ν_C=N_ 1604 cm^–1^. ES^+^MS (CH_3_CN): *m*/*z* 189.55 ([**2** – 3OTf]^3+^). Anal. Calcd for C_37_H_24_N_6_CrF_9_O_9_S_3_: C, 43.75; H, 2.38; N, 8.27. Found: C, 43.91; H, 2.54; N, 8.06%.

Syntheses of other heteroleptic complexes **3**–**9** follow very closely the methodology used for complex **2**; so, these are in the Supporting Information along with relevant characterization data.

### X-ray Crystallography

Single crystals of complexes **3**, **4**, **5**, **7**, and **8** were grown by diffusion of diethyl ether vapor into a saturated solution of each complex in acetonitrile. In each case, a suitable crystal was selected and attached to a glass fiber with Fomblin oil and mounted under a stream of cold N_2_ on a Rigaku Oxford Diffraction SuperNova diffractometer equipped with an Atlas-S2 CCD area detector (complexes **3** and **4**) or a Rigaku Oxford Diffraction Synergy-S diffractometer equipped with a hybrid pixel-array detector (complexes **5**, **7**, and **8**), in either case using Cu-K_α_ radiation (λ = 1.54184 Å) from a microfocus sealed tube source. Using Olex2 software,^82^ the structures were solved with the ShelXT structure solution program^83^ using intrinsic phasing and were refined with the ShelXL refinement package using least squares minimization.^84^ Crystallographic data, data collection/refinement parameters, and CSD deposition numbers are presented in the Supporting Information (Table S1). Specific issues for each structure associated with the disorder (often of anions) and treatment of solvents are discussed in individual CIFs.

### Photophysical Measurements

All photophysical measurements were undertaken with the complexes dissolved in 1 M HCl_(aq)_. This alters the nucleophilicity of the solvent, thereby decreasing the quantum yield of associative excited-state reactions (formation of seven-coordinate solvent species), which ultimately would lead to polypyridyl ligand substitution by solvent molecules.^1,71,85^ Ground-state absorption spectra were obtained at room temperature with a Shimadzu 1900-UV–visible spectrophotometer in quartz cuvettes with 1 cm path lengths. Quartz cuvettes (1 cm × 1 cm) with silicone septa seal screw caps were used for other solution photophysical measurements.

Emission spectra in solution were obtained with a Shimadzu RF-6000 instrument in the range of 200–900 nm. Emission spectra, emission quantum yields, and transient absorption spectra were obtained at 25 °C without deoxygenation; emission lifetime measurements were obtained at 25 °C with deoxygenation via Ar purging.

Luminescence quantum yields are calculated according to eq 1.
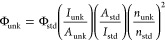
1

In this expression, Φ_unk_ and Φ_std_ are the emission quantum yields of the unknown and standard, respectively, under the conditions of the measurement. The quantities *I*_unk_ and *I*_std_ are the integrated emission intensities of the sample and the standard, respectively, over the range of 650–850 nm. The quantities *A*_unk_ and *A*_std_ are the absorbances of the sample and the standard, respectively, at an excitation wavelength of 320 nm. Care was taken to ensure that these are both close to 0.1. Finally, *n*_unk_ and *n*_std_ are the indices of refraction of the sample and standard solution, respectively. The luminescence emission quantum yield Φ_unk_ in 1 M HCl_(aq)_ was calculated relative to [Os(bpy)_3_]Cl_2_ in acetonitrile for which the absolute quantum yield Φ_std_ of 0.005 is known.^86^

An LP980 Edinburgh Instruments laser flash photolysis system was used for the collection of transient absorption spectra. The excitation source was a Nd:YAG Q-smart 450 Quantel Lasers instrument at 355 nm, and the pulse energy was ≤20 mJ. Singlet oxygen phosphorescence decays at 1270 nm were collected using a Hamamatsu H10330-45 NIR detector as previously described.^55^ Singlet oxygen quantum yields, Φ_Δ_, were obtained by comparing the luminescence intensity of singlet oxygen at 1270 nm photosensitized by the current set of complexes with that obtained from the reference [Ru(bpy)_3_]^2+^ at zero time in air-equilibrated D_2_O solution.^87^ Transient absorption decay traces of Cr(III) complexes at different oxygen concentrations were collected after purging the 1 M HCl solution for 20 min with argon or with oxygen and also in air-equilibrated solutions.^87^ Although the effect of pH on oxygen solubility cannot be neglected, it is not significant compared with the other main effects, such as temperature, pressure, and salinity. In our case, we used the same concentration of dissolved oxygen in air-saturated and oxygen-saturated 1 M HCl as in H_2_O, which are 0.27 and 1.27 mM, respectively,^87^ to calculate the oxygen quenching rate constant (as the ionic strength of 1 M HCl does not increase significantly as in 1 M H_2_SO_4_ compared to H_2_O).^88,89^

### Other Physical Methods

Infrared spectra were recorded on a Bruker α FTIR spectrometer; solid samples were measured by using the attenuated total reflection (ATR) technique. Electrospray ionization mass spectrometric measurements (ES-MS) were performed at room temperature in positive-ion mode on an Agilent 6130B single-quad mass spectrometer equipped with an analytical electrospray ion source and a quadrupole ion trap mass analyzer. The elemental analyses were measured with an EURO elemental analyzer from Euro Vector instruments.

## Results and Discussion

### Synthesis and Characterization

**Figure 1:**
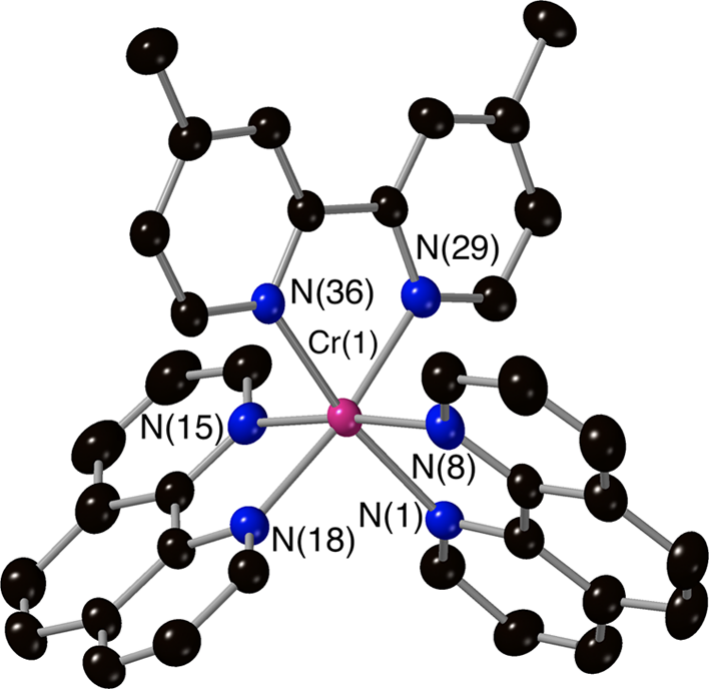
Molecular structure of the complex cation [Cr(Phen)_2_(4,4′-DMB)]^3+^ from crystallographic data with thermal ellipsoids drawn at the 50% probability level and only key atoms labeled (counterions and solvents have been removed for clarity).

**Figure 2:**
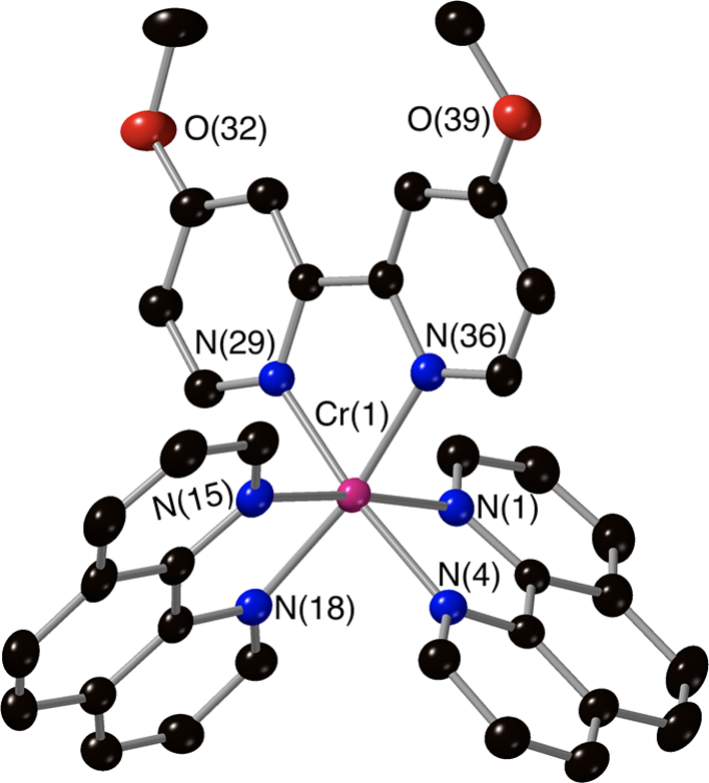
Molecular structure of the complex cation [Cr(Phen)_2_(4,4′-DMOB)]^3+^ from crystallographic data with thermal ellipsoids drawn at the 50% probability level and only key atoms labeled (counterions and solvents have been removed for clarity).

**Figure 3:**
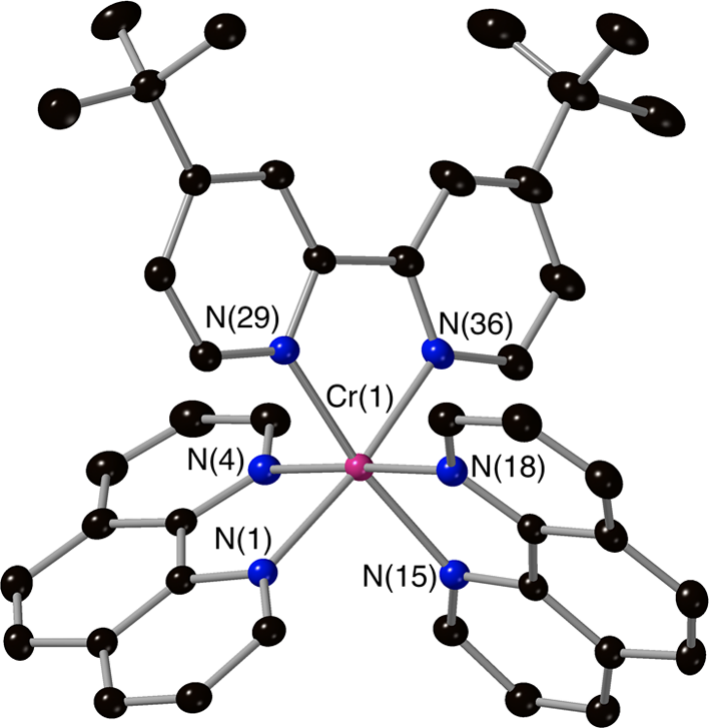
Molecular structure of the complex cation [Cr(Phen)_2_(4,4′-d^t^bpy)]^3+^ from crystallographic data with thermal ellipsoids drawn at the 50% probability level and with only key atoms labeled (counterions and solvents have been removed for clarity).

**Figure 4:**
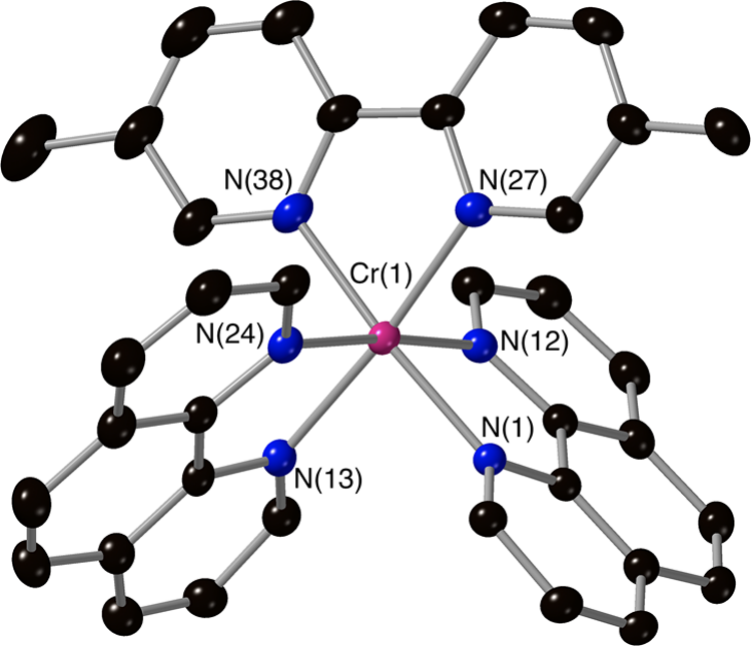
Molecular structure of the complex cation [Cr(Phen)_2_(5,5′-DMB)]^3+^ from crystallographic data with thermal ellipsoids drawn at the 50% probability level and with only key atoms labeled (counterions and solvents have been removed for clarity).

**Figure 5:**
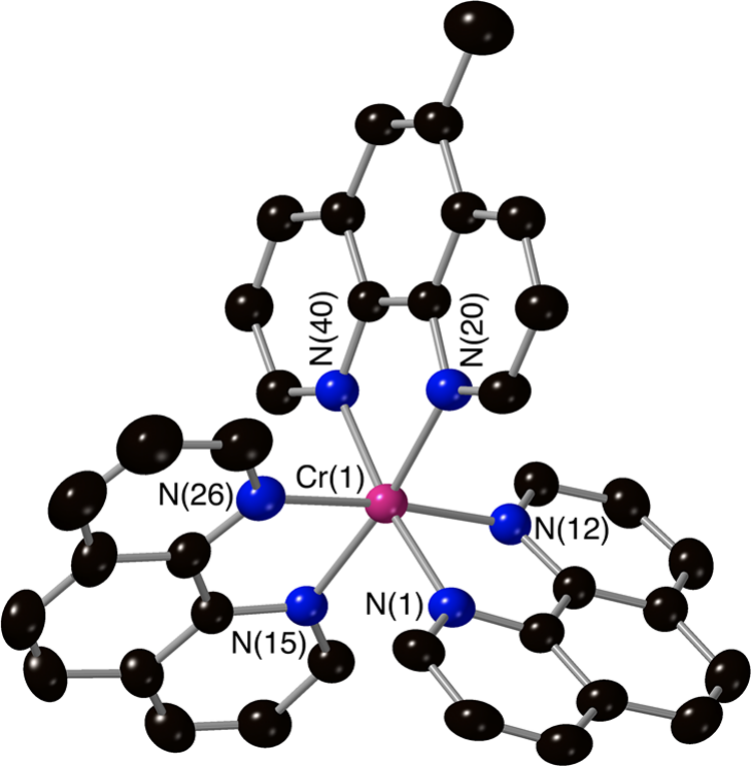
Molecular structure of the complex cation [Cr(Phen)_2_(5-Me-Phen)]^3+^ from crystallographic data with thermal ellipsoids drawn at the 50% probability level and with only key atoms labeled (counterions and solvents have been removed for clarity).

The homoleptic complex [Cr(Phen)_3_](OTf)_3_ (**1**) and heteroleptic complexes of the type [Cr(Phen)_2_(diimine)](OTf)_3_ [diimine = bpy (**2**), 4,4′-DMB (**3**), 4,4′-DMOB (**4**), 4,4′-d^t^bpy (**5**), 4,4′-dmcbpy (**6**), 5,5′-DMB (**7**), 5-Me-Phen (**8**), and 4,7-DMP (**9**)] were prepared using reported methodologies (see the Experimental section for details); all provided satisfactory electrospray mass spectra and C/H/N elemental analyses. Importantly, the mass spectra of heteroleptic complexes showed no evidence of undesired ligand-scrambled products. The crystal structures of complexes **3**, **4**, **5**, **7**, and **8** (Figures 1–5) confirm the mixed-ligand formulations, all displaying the expected slightly distorted octahedral metal complex core (associated with non-90° bite angles for the chelating ligands) and Cr–N bond distances in the range of 2.03–2.07 Å.

### Photophysical Studies

#### Absorption Spectroscopy

The typical absorption spectrum of Cr(III) complexes consists of three weak absorption bands (quartet–quartet) in the UV–visible spectral region, which result from the spin-allowed transitions from the ground state to the three excited quartet states ^4^T_2g_, a^4^T_1g_, and b^4^T_1g_, while the two spin-flip transitions in the red region of the spectrum are assigned to metal-centered ^4^A_2g_ → ^2^E_g_ and ^4^A_2g_ → ^2^T_1g_ transitions. In addition, more intense ligand-centered π–π* transitions occur in the UV region.^16^

**Figure 6:**
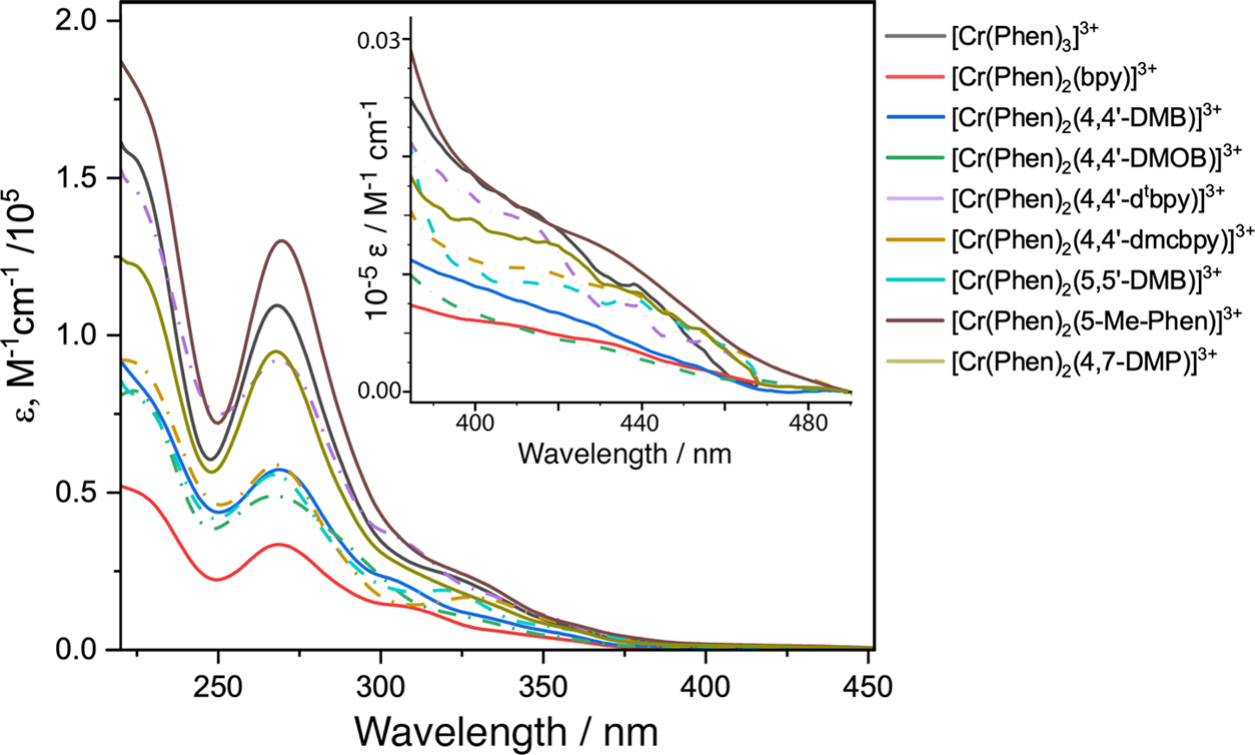
Electronic absorption spectra for the studied Cr(III) complexes in 1 M HCl_(aq)_ at room temperature. The inset shows the blue–visible spectral region on an enlarged scale.

**Figure 7:**
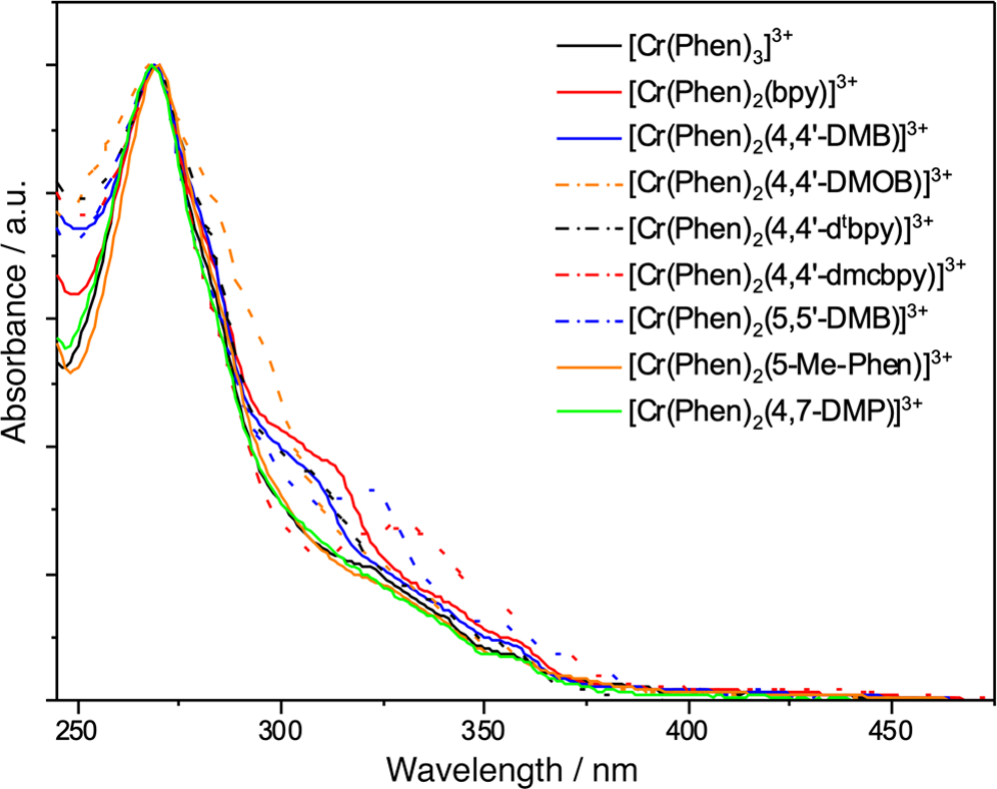
Normalized (against the ≈270 nm peak) electronic absorption spectra for the studied Cr(III) complexes in 1 M HCl_(aq)_ at room temperature.

The observed absorption spectra of the complexes shown in Figure 6 (and in Figure 7, which shows the same spectra normalized to the ligand-centered band at 269 nm) demonstrate clearly the effect of ligand variation on the absorption spectra. For example, complexes of the form [Cr(Phen)_2_(Xbpy)]^3+^ (where Xbpy is a substituted bipyridine ligand) show an absorption maximum at 269 nm and also a second band at 307, 308, 312, 323, and 331 nm for 4,4′-DMB, 4,4′-d^t^bpy, bpy, 5,5′-DMB, and 4,4′-dmcbpy, respectively. These absorption peaks are consistent with the observed peaks for the [Cr(bpy)_3_]^3+^ core, indicating transitions involving the two different ligand types in the heteroleptic complexes. These new complexes (containing one or more Phen ligands) show a broad absorption at around 270 nm, consistent with the UV–vis spectrum of [Cr(Phen)_3_]^3+^ as reported by Marusak *et al.*(90) The free Phen ligand also shows a broad absorption band at 269 nm in acetonitrile (MeCN). There are several successive shoulders at longer wavelength regions that are assigned to a combination of charge transfer transitions and ligand field transitions.^91^ In the visible region, for all complexes, we observe a shoulder of d–d origin around 430 nm that is assigned to the ^4^A_2g_ → ^4^T_2g_ electronic transitions with molar absorptivity of 392–1537 M^–1^ cm^–1^. The electronic absorption transitions of the studied Cr(III) complexes with the corresponding molar absorption coefficients are collected in Table 1.

**Table 1:** Room Temperature Electronic Absorption Spectra for Cr(III) Complexes **1–9** in 1 M Aqueous HCl

complex	λ_max_/nm (ε/M^–1^ cm^–1^)
**1**: [Cr(Phen)_3_](OTf)_3_	269 (113 694); 285 (69 238), sh; 321(24 016), sh; 340 (14 818), sh; 356 (8175); 387 (2299); 405 (1532), sh; 438 (766), sh; 455 (255), sh
**2**: [Cr(Phen)_2_(bpy)](OTf)_3_	269 (34 941); 285 (22 776), sh; 312 (13 044), sh; 338 (5677), sh; 355 (3446); 407 (540), sh; 430 (405), sh; 458 (135), sh
**3**: [Cr(Phen)_2_(4,4′-DMB)](OTf)_3_	269 (59 811); 284 (40 760), sh; 309 (20 601), sh; 338 (9082), sh; 357 (5205); 403 (886), sh; 420 (664), sh; 453 (221), sh
**4**: [Cr(Phen)_2_(4,4′-DMOB)](OTf)_3_	269 (50 098); 282 (40 760), sh; 295 (28 725), sh; 356 (4019); 425 (294), sh; 469 (35), sh
**5**: [Cr(Phen)_2_(4,4′-d^t^bpy)](OTf)_3_	269 (95 030); 283 (68 391), sh; 308 (34 323), sh; 338 (15 625), sh; 356 (8965); 410 (512), sh; 436 (256), sh
**6**:[Cr(Phen)_2_(4,4′-dmcbpy)](OTf)_3_	269 ( 61 029); 285 (35 108), sh; 330 (16 405), sh; 366 (5906); 413 (820), sh; 436 (656), sh; 454 (328), sh
**7**: [Cr(Phen)_2_(5,5′-DMB)](OTf)_3_	269 (57 497); 284 (35 888), sh; 323 (19 165), sh; 342 (9207), sh; 359 (6388); 418 (939), sh; 437 (563), sh; 454 (187), sh
**8**: [Cr(Phen)_2_(5-Me-Phen)](OTf)_3_	270 ( 135 013); 284 (87 889), sh; 329 (22 321), sh; 355 (9261); 433 (1029), sh
**9**: [Cr(Phen)_2_(4,7-DMP)](OTf)_3_	268 (98 313); 284 ( 60 057), sh; 341 (11 929), sh; 355 (7404); 399 (1234); 417 (1439), sh; 439 (822), sh; 454 (205), sh

#### Luminescence Spectroscopy

**Figure 8:**
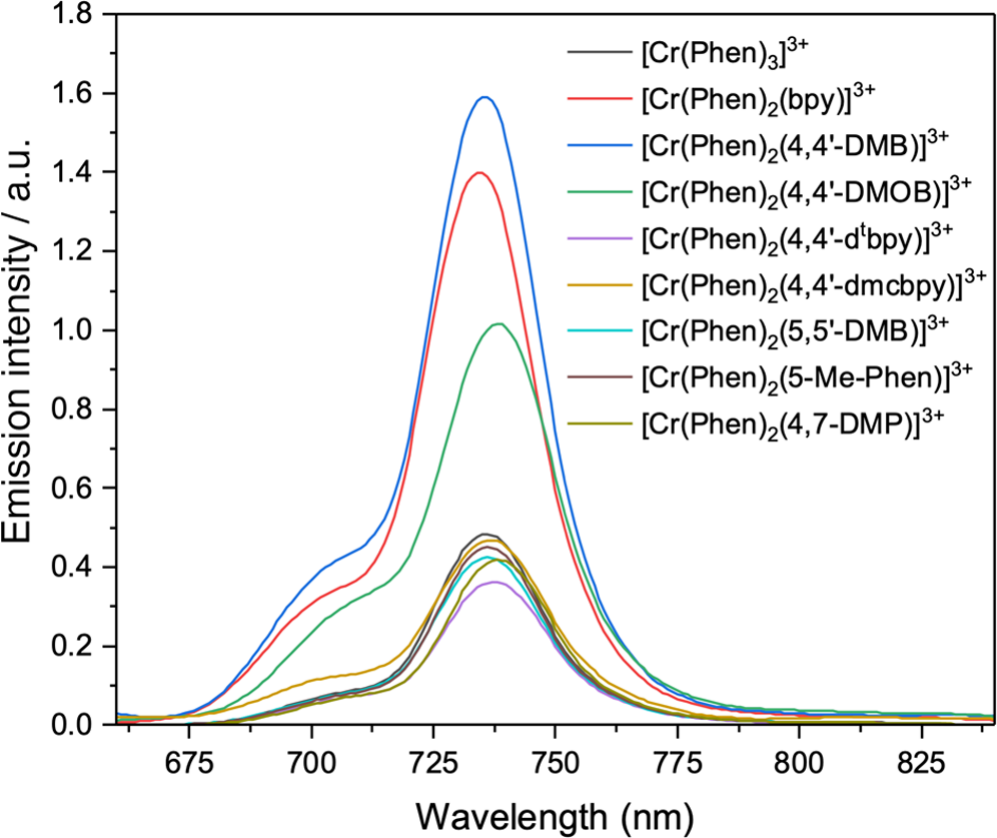
Emission spectra of the studied Cr(III) complexes in 1 M HCl_(aq)_ following excitation at 320 nm (absorbance = 0.1).

The luminescence spectra of Cr(III) polypyridyl complexes originate from the doublet states ^2^T_1g_ and ^2^E_g_, both of which arise from the electronic configuration (t_2g_)^3^.^91^ Insofar as the lifetimes of the two emission peaks (at ∼700 and ∼730 nm) are identical, these two excited states are thermally equilibrated.^90,92,93^Figure 8 shows that the wavelength of maximum luminescence emission for these complexes lies in all cases in the range of 734–738 nm and is largely invariant with any changes made to the ligands. The invariance observed for the maximum emission wavelength is common to Cr(III) complexes and indicates that the lowest energy excited state is insensitive to the ligand field, as would be the case for a ^2^E state, which arises solely from a spin change within the t_2g_ orbital set. By analogy with a large number of known emissive Cr(III) polypyridyl complexes, the main emission band seen at ∼730 nm is assigned to the ^2^E_g_ → ^4^A_2g_ transition, and the shoulder that occurs at ∼700 nm is assigned to the ^2^*T*_g_ → ^4^A_2g_ transition. Balzani and Hoffman et al. showed that the emission is not dependent on the excitation wavelength (312–450 nm), and the intersystem crossing (ISC) from ^4^T_2g_ to ^2^E_g_ takes place with almost unitary efficiency.^92^ The values reported in Table 2 are for *E*_00_ (equivalent to the Δ*G* stored in the excited state), where we have modified the observed maximum of the 0–0 vibronic transition (*E*_0_) with its width (Δν_0_,_1/2_) according to the expression^81^
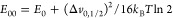
2For emissive MLCT species (commonly, Ru^II^, Os^II^, and Re^I^), one generally uses a Franck–Condon analysis to determine *E*_0_ and Δν_0_,_1/2_.^86,94,95^ Since the emissive features from the ^2^E → ^4^A ground state of these complexes are relatively narrow, we determine these quantities directly from the energy and width of the most intense band in the emission spectra. Calculation of *E*_00_ from the luminescence emission maxima in the range 734–738 nm results in a difference of 1.5 ± 0.5 kJ mol^–1^ compared to the value obtained from eq 2.

**Table 2:** Photophysical Properties of Cr(III) Polypyridyl Complexes^a^

complex	λ_em_/nm	*E*_00_, kJ mol^–1^	Φ_L_× 10^2^	τ_0_/ μs	*k*_q_/10^7^ M^–1^ s^–1^	Φ_Δ_	*P*_D_^O_2_^	*f*_Δ_^D^
1: [Cr(Phen)_3_](OTf)_3_	736	164.0	1.041	123.9	4.40	0.26	0.59	0.44
2: [Cr(Phen)_2_(bpy)](OTf)_3_	734	165.0	1.093	87.9	3.26	0.27	0.41	0.66
3: [Cr(Phen)_2_(4,4′-DMB)](OTf)_3_	735	165.0	1.484	104.8	3.69	0.30	0.51	0.59
4: [Cr(Phen)_2_(4,4′-DMOB)](OTf)_3_	738	164.0	1.203	99.9	5.27	0.39	0.60	0.65
5: [Cr(Phen)_2_(4,4′-d^t^bpy)](OTf)_3_	737	164.0	0.687	155.6	5.01	0.36	0.68	0.53
6:[Cr(Phen)_2_(4,4′-dmcbpy)](OTf)_3_	737	164.0	0.305	37.0	3.46	0.20	0.22	0.90
7: [Cr(Phen)_2_(5,5′-DMB)](OTf)_3_	736	164.0	1.301	90.0	3.65	0.32	0.44	0.72
8: [Cr(Phen)_2_(5-Me-Phen)](OTf)_3_	736	164.0	0.933	153.8	4.97	0.38	0.64	0.60
9: [Cr(Phen)_2_(4,7-DMP)](OTf)_3_	738	163.1	1.051	109.5	5.18	0.44	0.59	0.75

a λ_em_, wavelength of maximum emission; *E*_00_, energy of the excited state; Φ_L_, Luminescence quantum yield; τ_0_, excited state lifetime; *k*_q_, quenching by oxygen rate constant in air-equilibrated 1 M HCl; Φ_Δ_, singlet oxygen quantum yield; *P*_D_^O2^, fraction of the excited state quenched by oxygen; *f*_Δ_^D^, efficiency of singlet oxygen production, in D_2_O.

Table 2 shows that the luminescence quantum yields of all complexes are higher than those for the parent homoleptic complex [Cr(Phen)_3_](OTf)_3_, except for [Cr(Phen)_2_(4,4′-d^t^bpy)](OTf)_3_ and [Cr(Phen)_2_(4,4′-dmcbpy)](OTf)_3_, where the luminescence intensity drops to 2/3 and 1/3, respectively, relative to [Cr(Phen)_3_](OTf)_3_. The luminescence quantum yields of the substituted phenanthroline derivatives are similar to that of [Cr(Phen)_3_](OTf)_3_; on the other hand, dimethyl- and dimethoxy-substituted bipyridine heteroleptic complexes show higher luminescence quantum yields than [Cr(Phen)_3_](OTf)_3_.

#### Transient Absorption Measurements

**Figure 9:**
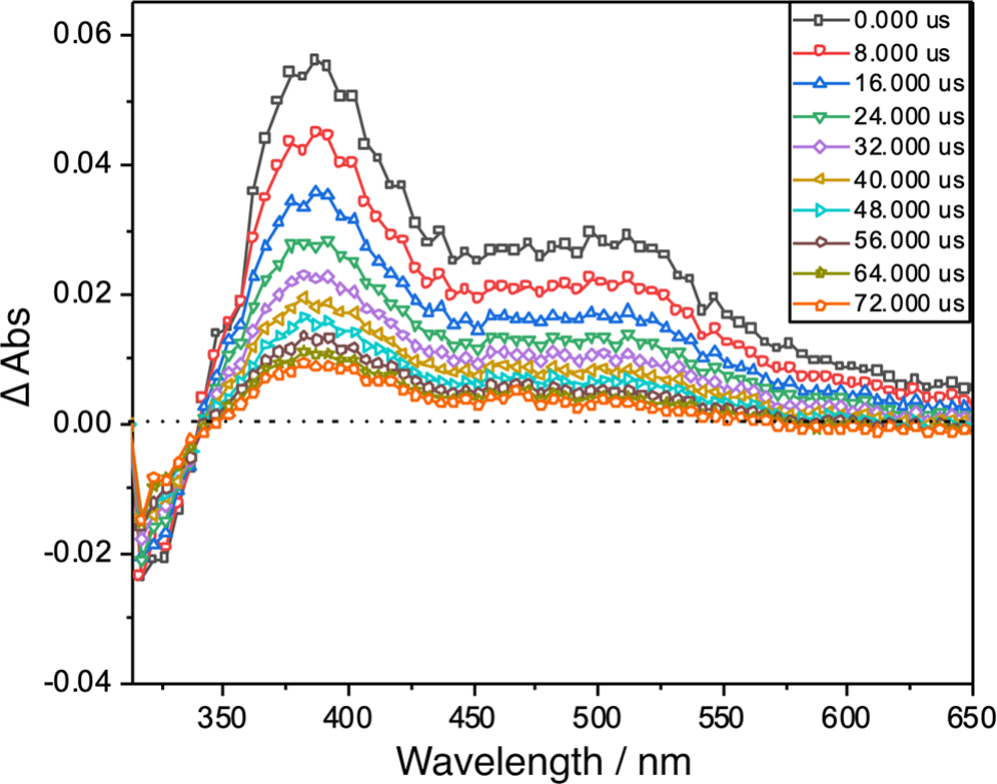
Transient absorption spectrum of [Cr(Phen)_2_(4,4′-DMOB)](OTf)_3_ at different time delays after excitation in air-equilibrated 1 M HCl.

Transient electronic absorption (TA) spectroscopy with time resolution from tens of picoseconds to milliseconds is considered a valuable tool in exploring absorption features once these molecules are electronically relaxed in the excited ^2^E excited states. On such time scales, it is well established that the ^2^E excited state of these complexes in fluid solution at room temperature is vibrationally cool and thermally equilibrated with the solvent.^96−103^ The symmetry of TA curves and the absence of abnormal peaks indicate that only one form is present in the excited state, and the formation of ion-separated species is absent. The TA spectrum of [Cr(Phen)_2_(4,4′-DMOB)](OTf)_3_, collected in 1 M HCl at room temperature, is shown in Figure 9. TA spectra for the remaining complexes in the series were collected under the same conditions and are presented in the Supporting Information (Figures S2–S9). The absorption below 350 nm is assigned to ground state depletion, while the strong absorption at longer wavelengths is assigned to charge transfer transitions originating from the ^2^E and ^2^T excited states.^81^ The excited state lifetime was obtained from the decay of the ^2^E decay traces at the wavelength of maximum absorption in the TA spectra. Table 2 shows that the lifetime of the excited state was found to correlate very well with the luminescence quantum yield of the current set of complexes, except for [Cr(Phen)_2_(4,4′-dmcbpy)](OTf)_3_, which shows the lowest excited state lifetime and luminescence quantum yield.

#### Oxygen Quenching Rate Constant, *k*_q_

The two low-lying ^2^T_1_ and ^2^E excited states, which are responsible for the two emission bands, have identical lifetimes, which indicate that these two states are in thermal equilibrium and are frequently represented simply as ^2^E;^90,92,93^ we adhere to this convenient and simple convention throughout the following discussion on quenching of the excited state by molecular oxygen. The observed lifetime of ^2^E is given by ^2^τ_obs_ = 1/^2^*k*, where ^2^*k* represents the rate constant of the individual decay modes. The lifetime of the emissive ^2^E states was measured in a deoxygenated, air-equilibrated, and oxygen-saturated 1 M HCl aqueous solution. The TA decay traces were found to fit well to a monoexponential relationship at different oxygen concentrations, and the resulting decay constants were used to determine the rate constants for oxygen quenching of the lowest excited state of the photosensitizer complexes, *k*_q_.

**Figure 10:**
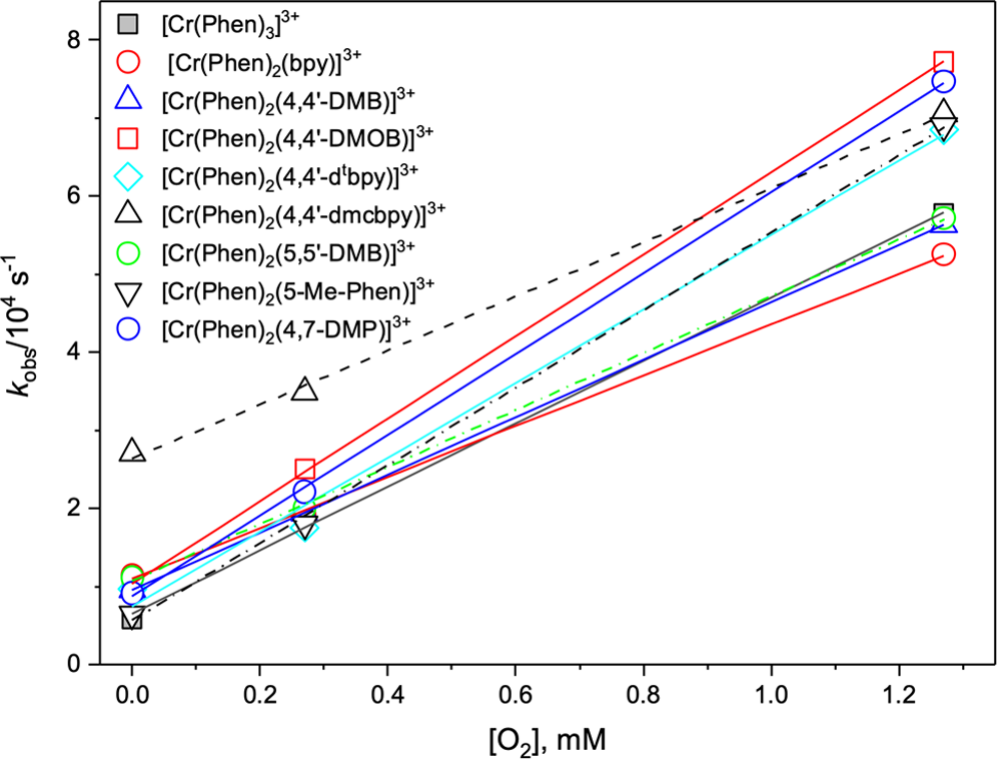
Stern–Volmer plot of the studied Cr(III) complexes in 1 M HCl at three different O_2_ concentrations.

The pseudo-first-order rate constant, *k*_obs_, of the excited ^2^E state decay is given by
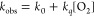
3where *k*_0_ (= 1/τ_0_) is the intrinsic first-order decay constant of the excited ^2^E state in the absence of the quencher (Figure 10).

#### Singlet Oxygen Measurements in D_2_O

The obtained singlet oxygen quantum yields arising from photosensitization by the current series of heteroleptic [Cr(Phen)_2_L]^3+^ complexes in D_2_O is correlated with (i) the fraction of the excited ^2^E states quenched by oxygen, *P*_D_^O_2_^; (ii) the quantum yield of formation of the excited ^2^E state population from the excited ^4^T states by intersystem crossing, Φ_isc_; and (iii) the efficiency of singlet oxygen production, *f*_Δ_^D^, through the following equation (the subscript D is used to represent the excited ^2^E state)
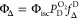
4The intersystem crossing efficiency from the excited ^4^T state to the lowest excited ^2^E states is claimed to be unity,^92^ and accordingly, eq 4 becomes
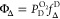
5The fraction of the excited ^2^E states quenched by oxygen can be obtained from the following eq 6 as follows
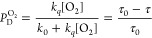
6where *k*_0_ is the intrinsic first-order decay constant of the ^2^E state (1/τ_0_) in the absence of oxygen; τ_0_ is the excited state lifetime in the absence of oxygen; and τ is the excited state lifetime in an air-equilibrated D_2_O solution. The obtained values of Φ_Δ_, *P*_D_^O2^ (using the oxygen concentration in air-equilibrated aqueous solution), and *f*_Δ_^D^ are compiled in Table 2.

The mechanism of oxygen quenching of the excited triplet states was first proposed by Gijzeman *et al.*(20) This was further developed by Garner and Wilkinson, who included intersystem crossing between the included charge transfer intermediates,^21^ and again later by Wilkinson and Abdel-Shafi, who include direct energy transfer in the singlet channel in competition with previous quenching pathways.^22,23^

**Scheme 1:**
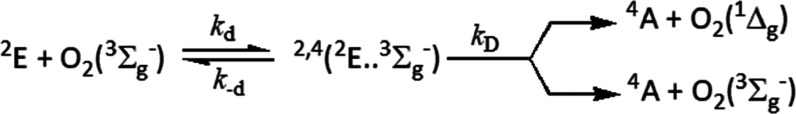
Application of Schmidt’s Model Modified with the Spin Statistical Parameters to the Quenching of the Excited ^2^E State of Cr(III) Complexes

We have previously successfully applied for the first time Schmidt’s model to oxygen quenching of the excited state of Ru(II), Os (II), Ir(III), and Re(I) complexes.^42−47^ The application of this model to the quenching of the excited ^2^E state of Cr(III) complexes is outlined as follows (Scheme 1). The excited ^2^E sensitizer and O_2_(^3^Σ_g_^–^) form an encounter complex of the form  with multiplicities *m* = 2 and 4 and with a diffusion-controlled rate constant *k*_d_. The encounter complexes  either dissociate back again with the rate constant *k*_–d_ or react forward to form the ground-state sensitizer (^4^A) and singlet oxygen O_2_(^1^Σ_g_^+^) or O_2_(^1^Δ_g_) through the doublet channel or the ground-state sensitizer ^4^A and ground-state oxygen O_2_(^3^Σ_g_^–^) through the quartet channel.

**Scheme 2:**
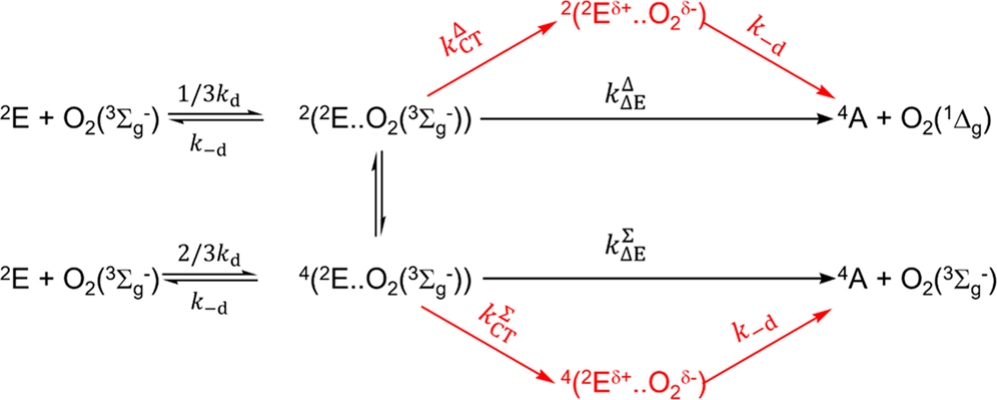
Spin Statistical Model Describing Energy *vs* Charge Transfer Pathways for the Quenching of the Excited ^2^E State of Cr(III) Complexes with Ground-State Molecular Oxygen

Schmidt *et al.*(24−30) have quantitatively identified the parameter *p*_CT_ that describes the balance between charge transfer (CT) and noncharge transfer (nCT) quenching pathways. The intriguing feature of this model is that the balance between CT and nCT can be obtained without knowledge of the oxidation potential of the photosensitizer. In this study, we adopt Schmidt’s model modified with the spin statistical parameters appropriate for the studied systems. In Scheme 2, the excited ^2^E state is quenched by the ground-state molecular oxygen, O_2_(^3^Σ_g_^–^), through the singlet channel with a rate constant of 1/3 *k*_d_ and through the doublet channel with the rate constant equal to 2/3*k*_d_, to form the excited complexes  and , respectively, and dissociation constant of *k*_–d_ in both channels. The singlet channel led to the formation of singlet oxygen O_2_(^1^Δ_g_) through an energy transfer pathway with the rate constant of *k*_Δ*E*_^Δ^ or charge transfer-assisted production of singlet oxygen through the charge transfer pathway with the rate constant of *k*_CT_^Δ^ forming the excited charge transfer complex , which dissociates with a rate of *k*_–d_ forming the ground-state complex ^4^A and singlet oxygen O_2_(^1^Δ_g_). On the other hand, in the energy dissipation channel (doublet channel), the encounter complex  is formed with a rate constant 2/3*k*_d_, leading finally to the ground state ^4^A of the Cr(III) complex and ground-state molecular oxygen either through energy-dependent, , or charge transfer, , pathways.

The overall rate constant *k*_D_ can be evaluated from *k*_q_ using eq 7 as follows
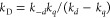
7and accordingly, the individual rate constants for the formation of O_2_(^1^Σ_g_^+^), , O_2_(^1^Δ_g_), , and O_2_(^3^Σ_g_^–^), , can be calculated from eqs 8–10 as follows
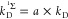
8
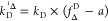
9
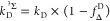
10The multiplicity-normalized rate constants *k*_D_^P^/*m* (i.e., ,  and ) depend on the excess energy Δ*E* for formation of O_2_(^1^Σ_g_^+^), O_2_(^1^Δ_g_) and O_2_(^3^Σ_g_^–^).

Internal conversion (IC) of the  nCT complexes occurs with rate constants of , , and  (where ) to lower-lying nCT complexes , , and , which dissociate to the respective products. The IC is ruled by the energy gap relation log(*k*_D_^P^/*m*) = *f*(Δ*E*), where Δ*E* is a polynomial equation of the fourth degree.^24−30^

In Scheme 2, intersystem crossing between the charge transfer encounter complexes is ignored, which is consistent with our previous suggestion.^104^ The rate constants for the formation of each O_2_ product state are additively composed of the nCT component *k*_D_^P^/*m* and the CT component *k*_CT_^P^/*m*. Thus, values of *k*_CT_^P^ are obtained from eqs 11–13.
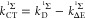
11
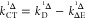
12
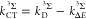
13The parameter *p*_CT_, which is the relative contribution of CT-mediated excited-state deactivation, is defined in eq 14 and is a quantitative measure of the balance between nCT and CT deactivation. *p*_CT_ is obtained for each sensitizer from *k*_Δ*E*_^P^ (polynomial equation given previously,^42^ and an intercept value of 6.80) and *k*_D_ (eq 7) with the knowledge of the excited-state ^2^E energy and quenching rate constant *k*_q_, respectively.
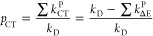
14Since  and  hold true, eqs 15 and 16 were used to evaluate the theoretical values of *f*_Δ_^D^ and *k*_q_ as follows^24−30^
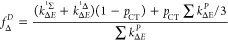
15
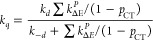
16

Based on the experimentally determined *f*_Δ_^D^ and *k*_q_, the proper values of *p*_CT_ were determined using eqs 15 and 16; by adjusting *p*_CT_ for each sensitizer until the discrepancy between the experimental and calculated values of *f*_Δ_^D^ and *k*_q_ is minimized, the optimal value of *p*_CT_ is attained. The obtained values of *k*_D_, *f*_Δ_^D^, and *k*_q_ are given in Table 3. The fitting parameters used were *k*_d_ = *k*_–d_ = 2.2 × 10^10^ s^–1^, and the same energy gap polynomial equation used before,^42^ with an intercept of 6.80.

**Table 3:** Experimental Rate Constants for Quenching of the Excited ^2^E State by Oxygen, *k*_*q*_, and the Efficiency of Singlet Oxygen Production, *f*_Δ_^D^, in D_2_O versus Their Corresponding Calculated Values and the Overall Rate Constants *k*_D_, *p*_CT_ Values, and Residuals (res) of Calculated *k*_q_ and *f*_Δ_^D^ Values

complex	log *k*_q_ exp	log *k*_q_ calc	*f*_Δ_^D^ exp	*f*_Δ_^D^ calc	*k*_D_/10^7^ s^–1^	*p*_CT_	res
1: [Cr(Phen)_3_](OTf)_3_	7.64	7.71	0.44	0.52	5.14	0.68	0.32
2: [Cr(Phen)_2_(bpy)](OTf)_3_	7.51	7.50	0.66	0.65	3.16	0.48	0.05
3: [Cr(Phen)_2_(4,4′-DMB)](OTf)_3_	7.57	7.57	0.59	0.60	3.73	0.56	0.02
4: [Cr(Phen)_2_(4,4′-DMOB)](OTf)_3_	7.72	7.66	0.65	0.55	4.57	0.64	0.27
5: [Cr(Phen)_2_(4,4′-d^t^bpy)](OTf)_3_	7.70	7.61	0.53	0.57	4.11	0.60	0.22
6: [Cr(Phen)_2_(4,4′-dmcbpy)](OTf)_3_	7.54	7.40	0.90	0.72	2.53	0.35	0.40
7: [Cr(Phen)_2_(5,5′-DMB)](OTf)_3_	7.56	7.51	0.72	0.64	3.22	0.49	0.21
8: [Cr(Phen)_2_(5-Me-Phen)](OTf)_3_	7.70	7.66	0.60	0.55	4.57	0.64	0.15
9: [Cr(Phen)_2_(4,7-DMP)](OTf)_3_	7.71	7.61	0.75	0.57	4.11	0.60	0.41

**Figure 11:**
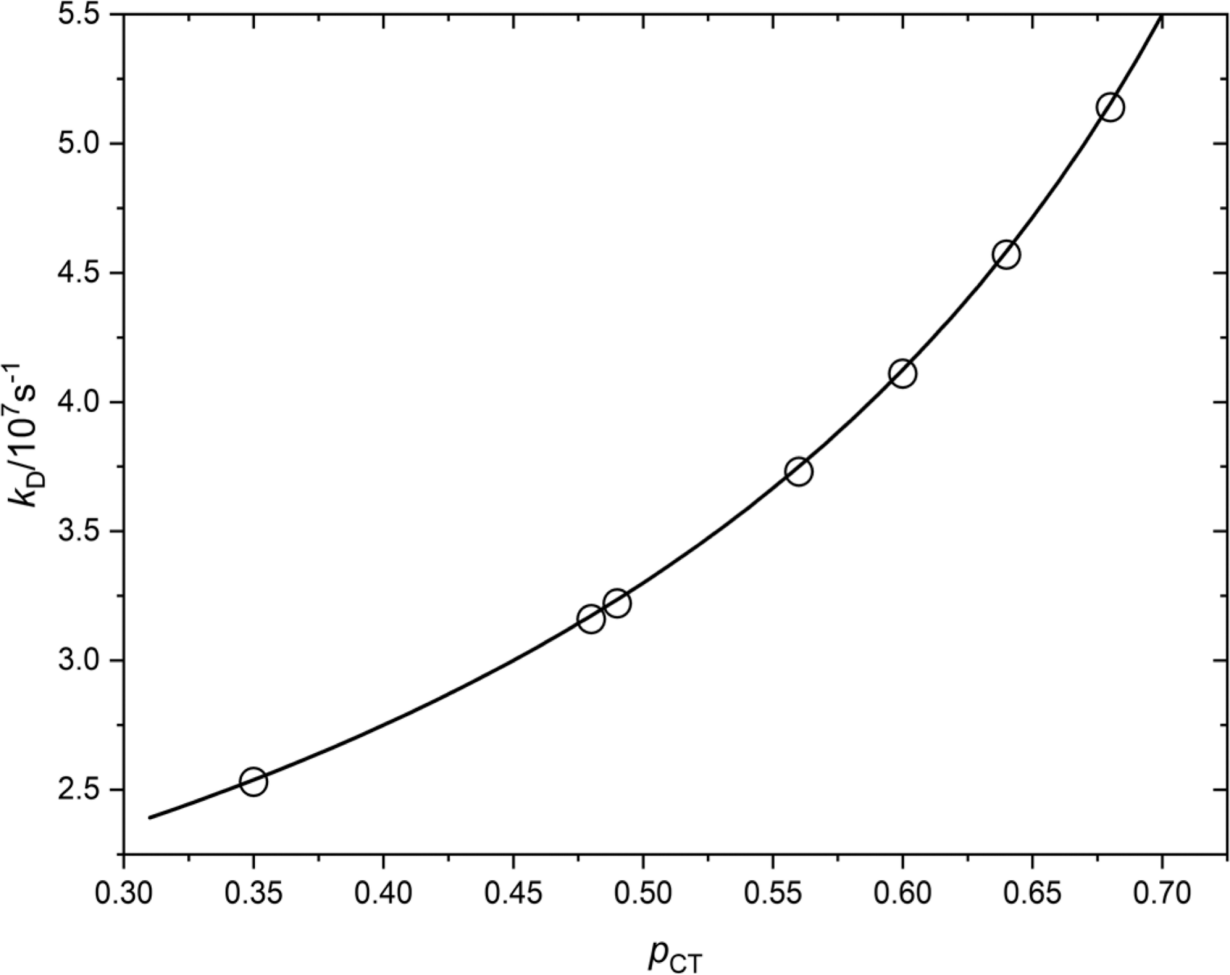
Dependence of the overall rate constant *k*_D_ of  complex deactivation during the quenching of the excited ^2^E states of the studied set of Cr(III) complexes by O_2_.

In contrast to our previous studies with ionic sensitizers, such as Ru(II) complexes,^42,44,56^ where the rate constant of back dissociation of the encounter complexes *k*_–d_ was taken as 3 × *k*_d_ × M, (where M is the unit mole per liter) or 2 × *k*_d_ × M in the case of Ir(III) and Re(I) complexes, the agreement between the calculated and experimental data presented in Table 3 is obtained when considering the rate constant of back dissociation of the encounter complexes, *k*_–d_ = *k*_d_ × M, which is similar to that used in the case of aromatic hydrocarbons in nonaqueous media.^22−25,105−107^Figure 11 shows the dependence of log(*k*_D_) on *p*_CT_. The nonlinear increase of log(*k*_D_) with *p*_CT_ is best described by the rearrangement of eq 14 (*k*_D_ = Σ*k*_Δ*E*_^P^/(1 – *p*_CT_)) with Σ*k*_Δ*E*_^P^ = 1.65 × 10^7^ s^–1^, which is much lower than observed for Ru(II) complexes for which Σ*k*_Δ*E*_^P^ = 2.4 × 10^9^ s^–1^.

Despite the fact that the proposed mechanism (Scheme 2) has a unique feature that allows evaluation of the balance between nCT and CT deactivation (*p*_CT_ parameter) without any knowledge of oxidation potential and solvent polarity, the variation of *p*_CT_ depends on the importance of the charge transfer interactions in the excited state complexes , which depend on the oxidation potential of the sensitizer and the solvent polarity. The evaluation of the oxidation potential of Cr(III) complexes was difficult to obtain in this study and was not reported previously^81^ as they are outside of the acetonitrile solvent oxidation window, and hence, the evaluation of the fractional dependence of the free energy of activation on the driving force of charge transfer in the case of the current set of Cr(III) complexes was not possible.

It is worth noting that the oxygen quenching rate constant for these Cr(III) complexes is at least 2 orders of magnitude slower than that for the corresponding Ru(II) complexes. For comparison, the oxygen quenching rate constant photosensitized by [Ru(Phen)_3_]^2+^ is 4.6 × 10^9^ M^–1^ s^–1,^^56^ whereas the value drops to 4.4 × 10^7^ M^–1^ s^–1^ for [Cr(Phen)_3_]^3+^. On the other hand, the singlet oxygen quantum yield is approximately the same for both sensitizers. It has been claimed that the lower oxygen quenching rate constant in the case of Cr(III) complexes arises from the different nature of the excited state, being metal-centered in the case of Cr(III) complexes, in contrast to MLCT for Ru(II) complexes.^108^ However, the approximate similarity of the efficiency of singlet oxygen production and singlet oxygen quantum yield produced by both sensitizers and the huge difference in the quenching rate constants can be explained as arising from the higher dissociation rate constants for the Ru(II) complexes (*k*_*-*d_ = 3*k*_d_). Therefore, the slower oxygen quenching rate constant for Cr(III) complexes compared to Ru(II) complexes can be assigned to several factors, such as the ligand shield effect that restricts the overlap between the ^2^E orbitals and ground-state orbitals of molecular oxygen, the nature of the excited state, and/or the spin nature of the interacting species.^70^

## Conclusions

The photophysical properties of a series of Cr(III) complexes are reported. Despite the near-constant excited state energy, *E*_0–0_, the luminescence quantum yields and excited state lifetimes were found to vary considerably with the structures of the complexes. The mechanism of the excited ^2^E state quenching by oxygen was studied in detail based on the spin statistical kinetic model shown in Scheme 2. This model predicted the balance between charge transfer and noncharge transfer pathways in the quenching process by the parameter, *p*_CT_. The unique feature of this model is that it allows the prediction of the contribution of the charge transfer interactions without knowledge of the oxidation potential of the sensitizer. We were able to successfully apply the kinetic model and obtained variable values for *p*_CT_ for sensitizers of different structures. The model was also successful in reproducing values of the quenching rate constants, *k*_q_, and the efficiency of singlet oxygen, *f*_Δ_^D^, photosensitized by the current set of Cr(III) complexes, with good agreement between the experimentally obtained values and those calculated by the model.

## Data Availability

Additional data can be found in the Supporting Information file.

## Supporting Information Available

Crystallographic data and data collection/refinement parameters are presented in Table S1. The ground and excited state reduction potential for Cr(III) polypyridyl complexes are presented in Table S2. Comparison of cyclic voltammograms for all Cr(III) polypyridyl complexes in 0.1 M [nBu_4_N][PF_6_] acetonitrile solution are presented in Figure S1. The normalized emission spectra for Cr(III) complexes in 1 M HCl(aq) following excitation at 320 nm are presented in Figure S2. TA spectra for the remaining Cr(III) complexes in the series were collected in 1 M HCl at room temperature and are presented as shown in Figures S3–S10. Synthetic details and characterization data for complexes **3**–**9** are also included (PDF)
