# Supplementary material for: Mechanism of Oxygen Quenching of the Excited States of Heteroleptic Chromium(III) Phenanthroline Derivatives
Source: Inorg Chem. 2023 Sep 18;62(39):16101–13. doi: 10.1021/acs.inorgchem.3c02343 (PMC10548418; doi:10.1021/acs.inorgchem.3c02343)
Supplement: Supplementary file 1 — ic3c02343_si_001.pdf [file ic3c02343_si_001.pdf]

Supporting Information

Mechanism of Oxygen Quenching of The Excited States of Heteroleptic Chromium (III) Phenanthroline Derivatives.

Ahmed M. M. Alazaly[a,b], Guy J. Clarkson,[b] Michael D. Ward[b] and Ayman A. Abdel-Shafi[a*]*

[a] Department of Chemistry, Faculty of Science, Ain Shams University, 11566, Abbassia, Cairo, Egypt.

[b] Department of Chemistry, University of Warwick, Coventry CV4 7AL, UK.

Corresponding Author

Ayman A. Abdel-Shafi – Department of Chemistry, Faculty of Science, Ain Shams University, 11566, Abbassia, Cairo, Egypt; Email: aaashafi@sci.asu.edu.eg

Michael D. Ward – Department of Chemistry, University of Warwick, Coventry CV4 7AL, UK; E-mail: m.d.ward@warwick.ac.uk

Contents:

Table S1. Crystallographic, data collection, and refinement details for the five structures. 4

Table S2. Ground and excited state redox potentials for the Cr(III) complexes. 5

Figure S1. Comparison of cyclic voltammograms for all Cr(III) polypyridyl complexes (0.1M $[nBu_4N][PF_6]$ in acetonitrile). Arrows indicate the starting point and direction for each voltammogram. 6

Figure S2. Normalized emission spectra for Cr(III) complexes in 1 M HCl(aq) following excitation at 320 nm. 7

Figure S3. TA spectrum of $[Cr(Phen)_3](OTf)_3$ in air-equilibrated 1 M HCl at a range of different time delays.. 8

Figure S4. TA spectrum of $[Cr(Phen)_2(bpy)](OTf)_3$ in air-equilibrated 1 M HCl at a range of different time delays. 8

Figure S5. TA spectrum of $[Cr(Phen)_2(4,4'\text{-DMB})](OTf)_3$ in air-equilibrated 1 M HCl at a range of different time delays.. 9

Figure S6. TA spectrum of $[Cr(Phen)_2(4,4'\text{-d}^t\text{bpy})](OTf)_3$ in air-equilibrated 1 M HCl at a range of different time delays.. 9

Figure S7. TA spectrum of $[Cr(Phen)_2(4,4'\text{-dmcbpy})](OTf)_3$ in air-equilibrated 1 M HCl at a range of different time delays. 10

Figure S8. TA spectrum of $[Cr(Phen)_2(5,5'\text{-DMB})](OTf)_3$ in air-equilibrated 1 M HCl at a range of different time delays.. 10

Figure S9. TA spectrum of $[Cr(Phen)_2(5\text{-Me-Phen})](OTf)_3$ in air-equilibrated 1 M HCl at a range of different time delays. 11

Figure S10. TA spectrum of $[Cr(Phen)_2(4,7\text{-DMP})](OTf)_3$ in air-equilibrated 1 M HCl at a range of different time delays. 11

Additional experimental information: preparation and analytical data for complexes **3** – **9**……………………………………………………………………………………12

REFERENCES ……………………………………………………………………………..15

Table S1. Crystallographic, data collection, and refinement details for the five structures.

Complex	**3•(MeCN)$_2$•(H$_2$O)$_{0.5}$**	**4•(MeCN)$_{1.05}$(Et$_2$O)$_{0.25}$(H$_2$O)$_{0.15}$**	**5•(MeCN)$_{3.25}$(H$_2$O)$_{0.25}$**	**7•(MeCN)$_2$**	**8•(H$_2$O)$_{0.35}$**
Empirical formula	$C_{43}H_{34}CrF_9N_8O_{9.5}S_3$	$C_{42.1}H_{33.65}CrF_9N_{7.05}O_{11.4}S_3$	$C_{51.5}H_{49.75}CrF_9N_{9.25}O_{9.25}S_3$	$C_{43}H_{34}CrF_9N_8O_9S_3$	$C_{40}H_{26}CrF_9N_6O_{9.35}S_3$
Formula weight	1133.96	1139.89	1265.43	1125.96	1058.65
Temperature/K	150(2)	150(2)	100(2)	100(2)	100(2)
Crystal system	triclinic	triclinic	triclinic	monoclinic	tetragonal
Space group	*P*-1	*P*-1	*P*-1	*C*2/c	$I4_1/a$
a/Å	12.9284(3)	13.11083(17)	11.80760(10)	24.5523(2)	36.3868(3)
b/Å	13.8883(5)	13.2659(2)	16.02070(10)	10.48168(10)	36.3868(3)
c/Å	14.7120(5)	15.45085(19)	16.73970(10)	36.8920(3)	12.6176(2)
α/°	98.455(3)	100.6525(12)	96.5170(10)	90	90
β/°	108.948(3)	106.0728(11)	110.6360(10)	102.0040(8)	90
γ/°	99.932(2)	98.6529(12)	97.1890(10)	90	90
Volume / Å^3	2401.85(13)	2479.24(6)	2897.45(4)	9286.53(14)	16705.7(4)
Z	2	2	2	8	16
ρ_{calc} / g.cm^{-3}	1.568	1.527	1.450	1.611	1.684
μ/mm^{-1}	4.068	3.968	3.435	4.195	4.614
F(000)	1154.0	1160.0	1301.0	4584.0	8566.0
Crystal size/mm^3	0.2 × 0.14 × 0.12	0.3 × 0.3 × 0.1	0.2 × 0.2 × 0.02	0.16 × 0.06 × 0.02	0.16 × 0.1 × 0.06
Data/restraints/ parameters	16333/433/813	9970/148/751	12414/86/841	9934/159/754	8944/111/682
GOOF on F^2	1.020	1.049	1.028	1.038	1.019
R_1, wR_2 [$I \geq 2\sigma(I)$]	0.0761, 0.2176	0.0536, 0.1484	0.0528, 0.1406	0.0518, 0.1342	0.0666, 0.1597
R_1, wR_2 [all data]	0.0834, 0.2259	0.0550, 0.1505	0.0551, 0.1424	0.0545, 0.1362	0.0806, 0.1684
Largest diff. peak/hole / e Å^{-3}	0.97/-1.15	0.72/-0.62	1.21/-0.93	0.94/-0.71	0.73/-0.56

Table S2. Ground and excited state redox potentials for the Cr(III) complexes.

($E_{1/2}$ vs Fc^{+}/Fc, V)[a]

Name	3+/2+	2+/1+	1+/0	0/1-	* 3+/2+
1: $[Cr(Phen)_3](OTf)_3$	-0.74 (-0.65)[1]	-1.25 (-1.17)[1]	-1.79 (-1.71)[1]		+0.96 (+1.05)[1]
2: $[Cr(Phen)_2(bpy)](OTf)_3$	-0.40	-0.93	-1.49		+1.31
3: $[Cr(Phen)_2(4,4'\text{-DMB})](OTf)_3$	-0.57	-1.08	-1.51		+1.14
4: $[Cr(Phen)_2(4,4'\text{-DMOB})](OTf)_3$	-0.52	-1.05	-1.58		+1.18
5: $[Cr(Phen)_2(4,4'\text{-d}^t\text{bpy})](OTf)_3$	-0.76	-1.26	-1.60	-1.83	+0.94
6: $[Cr(Phen)_2(4,4'\text{-dmcbpy})](OTf)_3$	-0.41 (-0.42)[1]	-1.0 (-1.01)[1]	-1.59 (-1.61)[1]	-1.87 (-1.90)[1]	+1.29 (+1.28)[1]
7: $[Cr(Phen)_2(5,5'\text{-DMB})](OTf)_3$	-0.71	-1.22	-1.56	-1.80	+0.99
8: $[Cr(Phen)_2(5\text{-Me-Phen})](OTf)_3$	-0.75	-1.26	-1.58	-1.82	+0.95
9: $[Cr(Phen)_2(4,7\text{-DMP})](OTf)_3$	-0.83	-1.35	-1.59	-1.89	+0.87

[a] Conditions for cyclic voltammetry of Cr complexes. Solvent, MeCN; electrolyte, 0.1 M $TBAPF_6$; WE, Pt; CE, Pt wire; scan rate, 0.1 V/s. Redox potential values that have been previously reported in ref. 1 are shown in red for comparison.

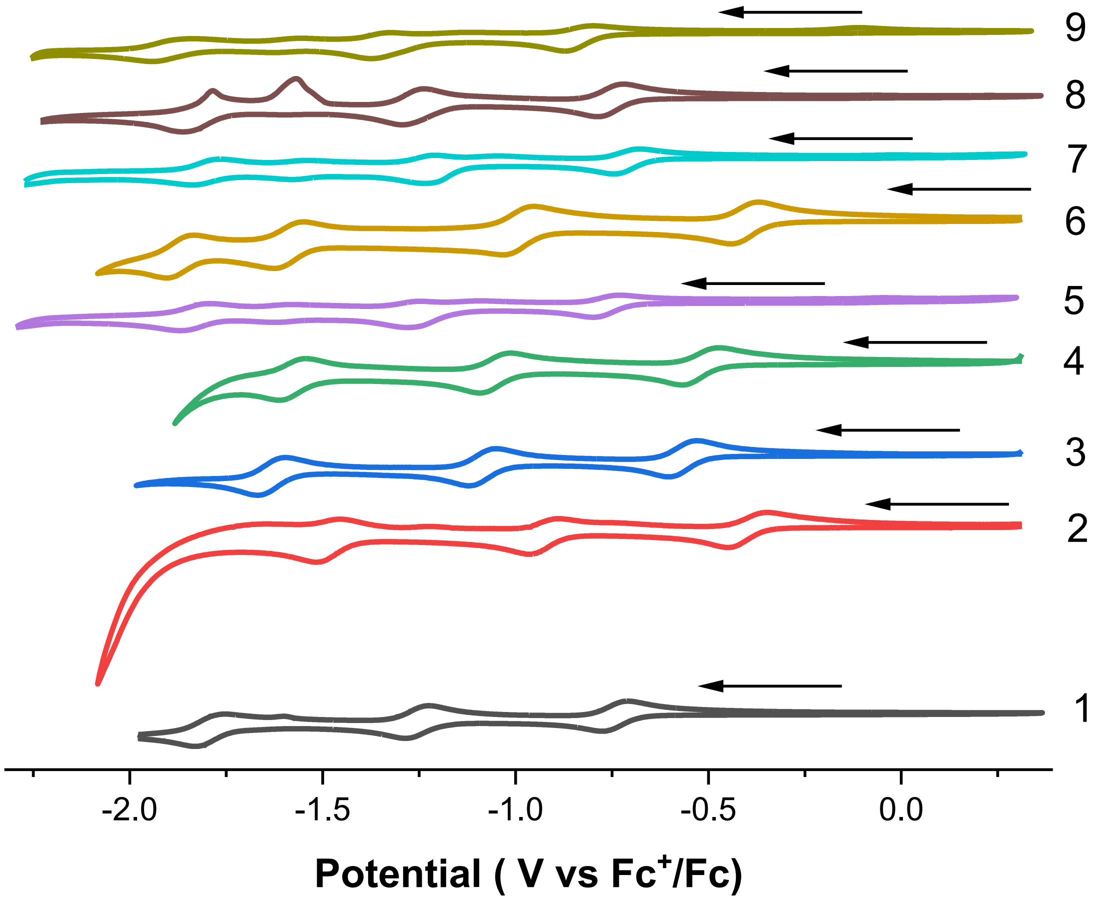

Figure S1. Comparison of cyclic voltammograms for all Cr(III) polypyridyl complexes (0.1M [nBu_4N][PF_6] in acetonitrile). Arrows indicate the starting point and direction for each voltammogram.

Normalized Emission Spectra

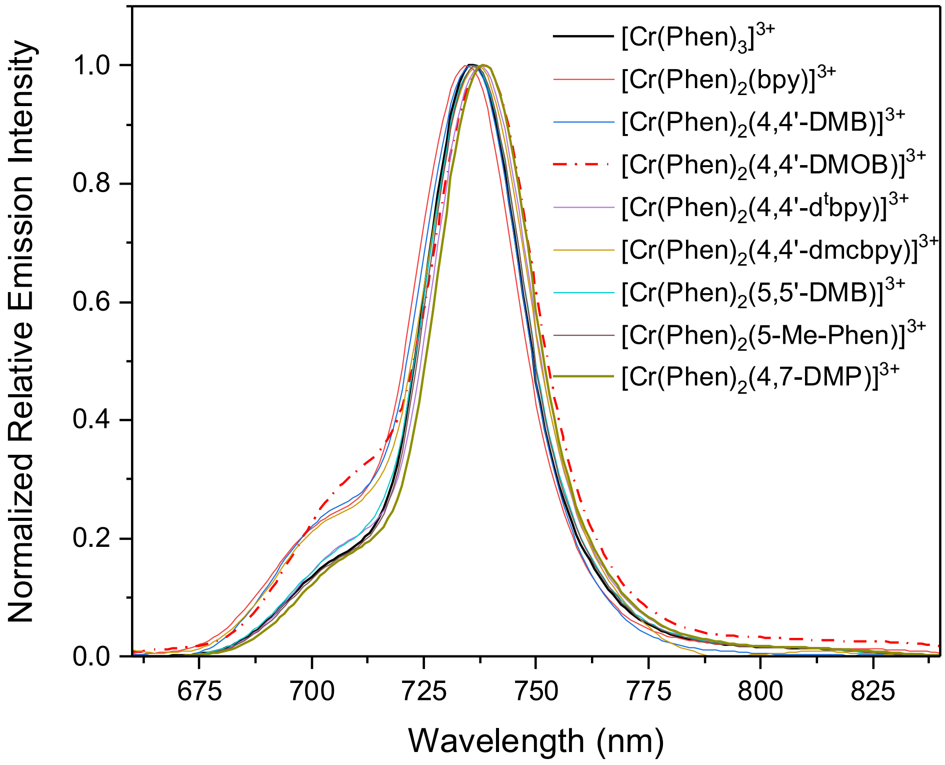

Figure S2. Normalized emission spectra for Cr(III) complexes in 1 M HCl(aq) following excitation at 320 nm.

Transient absorption spectra of the Co(III) complexes

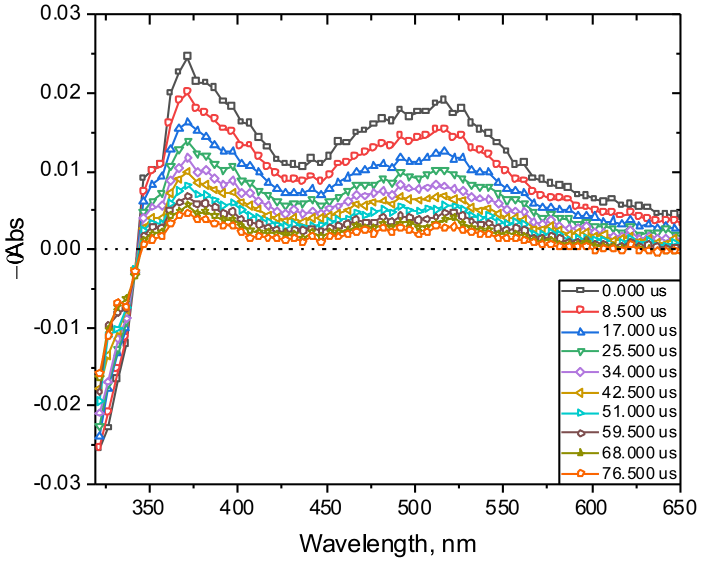

Figure S3. TA spectrum of [Cr(Phen)3](OTf)3 in air-equilibrated 1 M HCl at a range of different time delays.

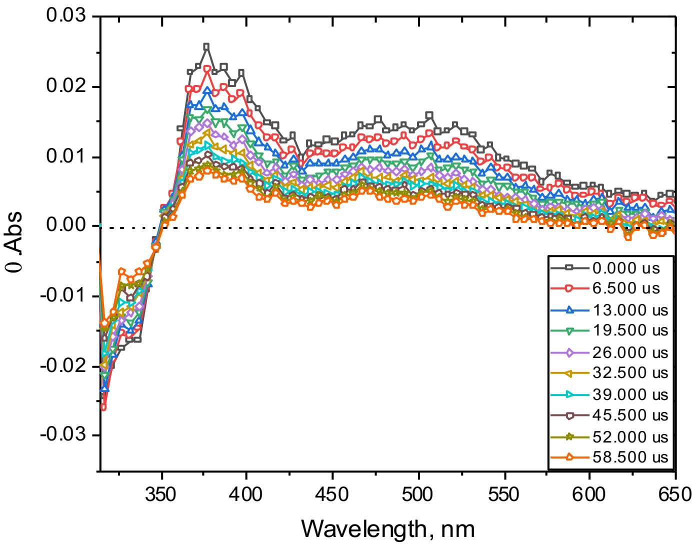

Figure S4. TA spectrum of [Cr(Phen)2(bpy)](OTf)3 in air-equilibrated 1 M HCl at a range of different time delays.

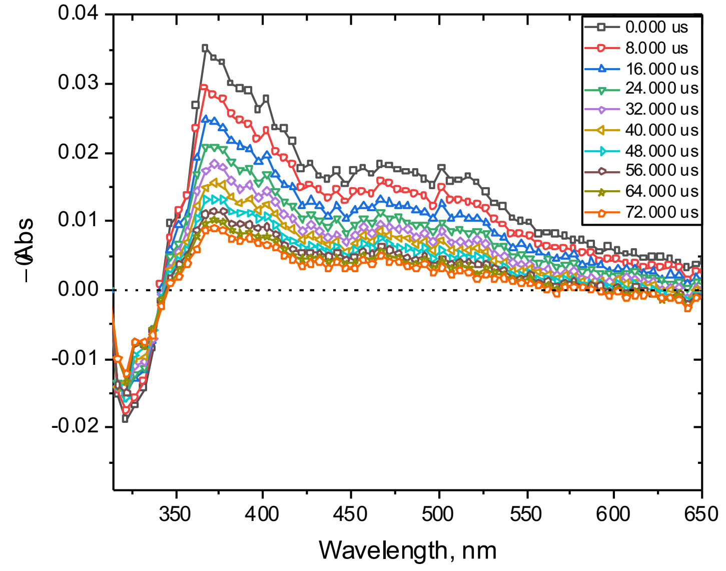

Figure S5. TA spectrum of [Cr(Phen)$_2$(4,4'-DMB)](OTf)$_3$ in air-equilibrated 1 M HCl at a range of different time delays.

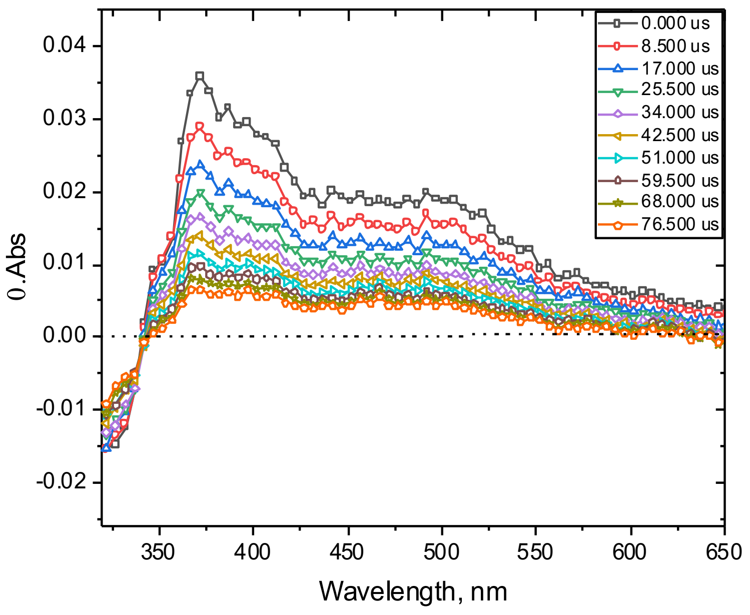

Figure S6. TA spectrum of [Cr(Phen)$_2$(4,4'-d^{t}bpy)](OTf)$_3$ in air-equilibrated 1 M HCl at a range of different time delays.

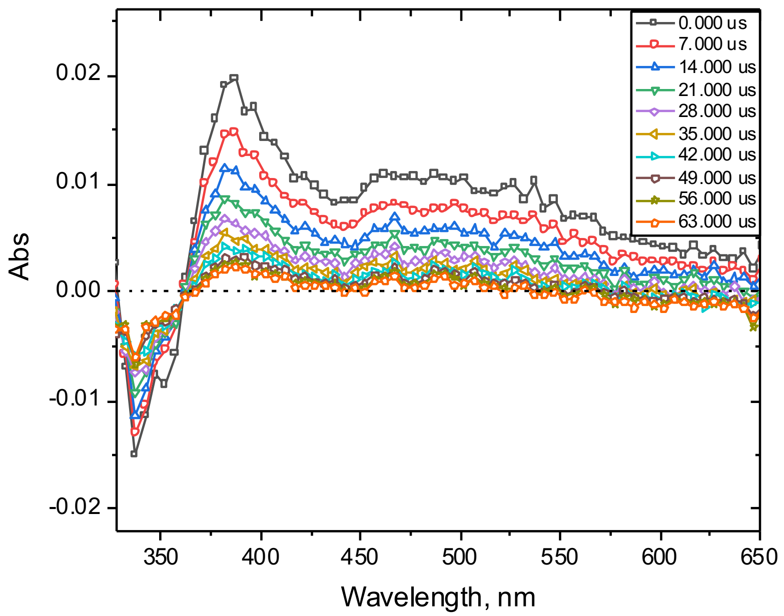

Figure S7. TA spectrum of [Cr(Phen)$_2$(4,4'-dmcbpy)](OTf)$_3$ in air-equilibrated 1 M HCl at a range of different time delays.

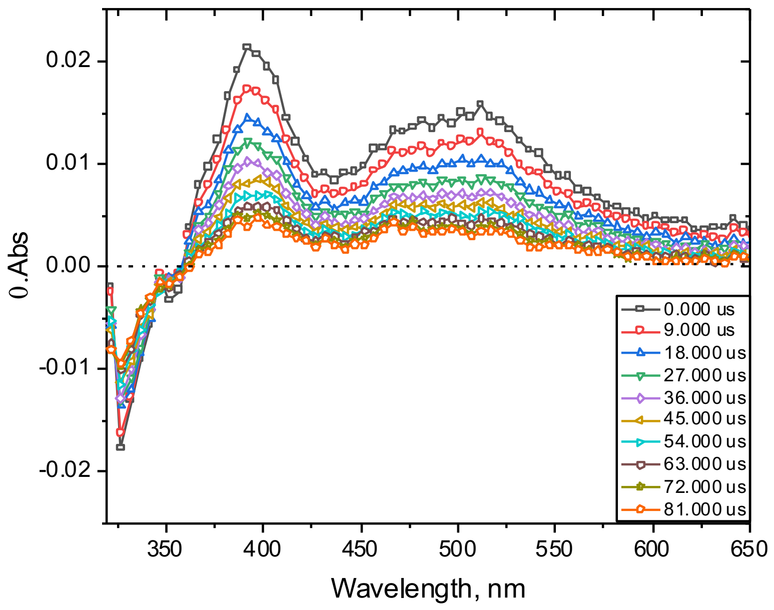

Figure S8. TA spectrum of [Cr(Phen)$_2$(5,5'-DMB)](OTf)$_3$ in air-equilibrated 1 M HCl at a range of different time delays.

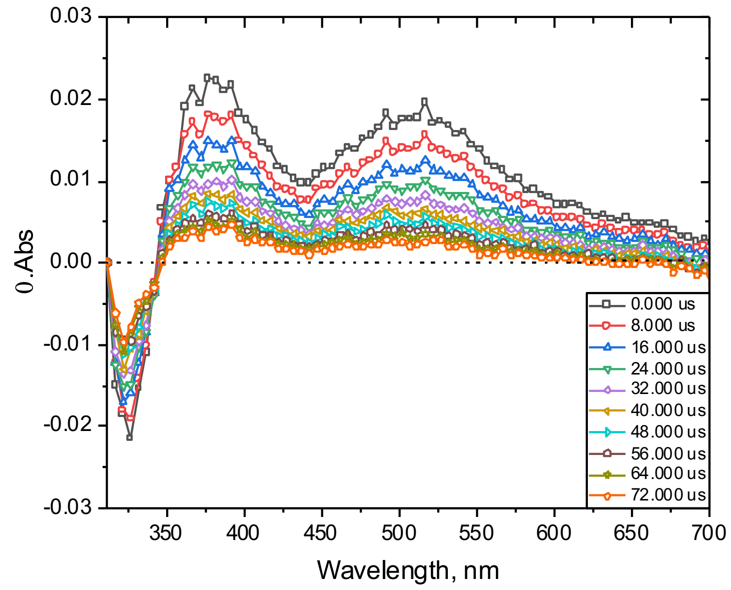

Figure S9. TA spectrum of [Cr(Phen)$_2$(5-Me-Phen)](OTf)$_3$ in air-equilibrated 1 M HCl at a range of different time delays.

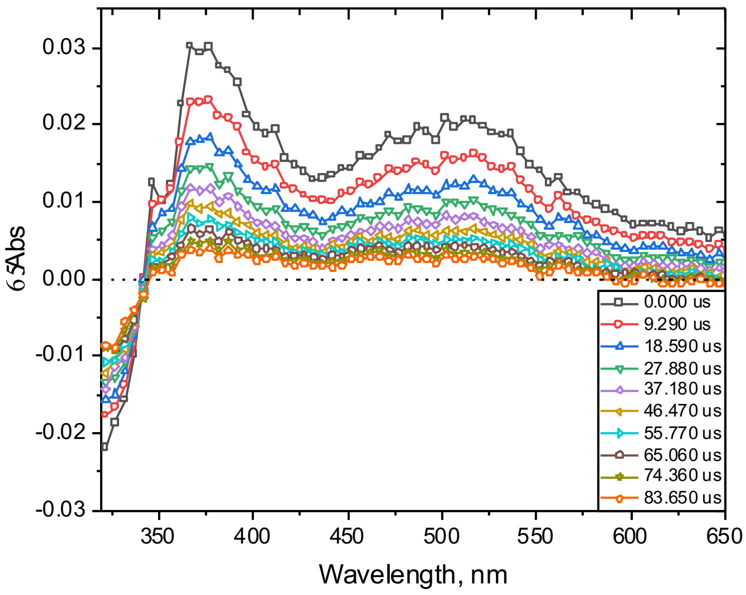

Figure S10. TA spectrum of [Cr(Phen)$_2$(4,7-DMP)](OTf)$_3$ in air-equilibrated 1 M HCl at a range of different time delays.

Additional experimental information: preparation and analytical data for complexes 3 – 9

Synthesis of [Cr(Phen)$_2$(4,4'-DMB)](OTf)$_3$ (3)

[Cr(Phen)$_2$(CF$_3$SO$_3$)$_2$](CF$_3$SO$_3$) (0.8 g, 0.93 mmol) and 4,4'-DMB (429 mg, 2.33 mmol) were suspended in CH$_2$Cl$_2$ (80 ml). The reaction mixture was heated to reflux for 30 h and a yellow precipitate was formed. This was separated by filtration, washed with CH$_2$Cl$_2$ (2 x 10 ml), and dried in vacuo. [Cr(Phen)$_2$(4,4'-DMB)](OTf)$_3$ was isolated as a yellow powder (0.73 g, 91 %). IR: $\nu_{C=N}$ 1618 cm^{-1}. ES$^+$MS (CH$_3$CN): *m/z* 198.90 ([**3** – 3OTf]$^{3+}$). Anal. Calcd. for $C_{39}H_{28}N_6CrF_9O_9S_3$: C, 44.87; H, 2.70; N, 8.05. Found: C, 45.10; H, 2.56; N, 7.85 %.

Synthesis of [Cr(Phen)$_2$(4,4'-DMOB)](OTf)$_3$ (4)

[Cr(Phen)$_2$(CF$_3$SO$_3$)$_2$](CF$_3$SO$_3$) (0.8 g, 0.93 mmol) and 4,4'-DMOB (500 mg, 2.31 mmol) were suspended in CH$_2$Cl$_2$ (50 ml). The reaction mixture was heated to reflux for 30 h and a yellow precipitate was formed. This was separated by filtration, washed with CH$_2$Cl$_2$ (2 x 10 ml), and dried in vacuo. [Cr(Phen)$_2$(4,4'-DMOB)](OTf)$_3$ was isolated as a yellow powder (0.78 g, 78 %). IR: $\nu_{C=N}$ 1618 cm^{-1}. ES$^+$MS (CH$_3$CN): *m/z* 209.55 ([**4** – 3OTf]$^{3+}$). Anal. Calcd. for $C_{39}H_{28}N_6CrF_9O_{11}S_3$: C, 43.54; H, 2.62; N, 7.81. Found: C, 43.75; H, 2.62; N, 7.57 %.

Synthesis of [Cr(Phen)$_2$(4,4'-d^{t}bpy)](OTf)$_3$ (5)

[Cr(Phen)$_2$(CF$_3$SO$_3$)$_2$](CF$_3$SO$_3$) (0.74 g, 0.86 mmol) and 4,4'-d^{t}bpy (335 mg, 1.25 mmol) were suspended in CH$_2$Cl$_2$ (60 ml). The reaction mixture was heated to reflux. Over 5 days a yellow precipitate formed which was separated by filtration, washed with CH$_2$Cl$_2$ (2 x 10 ml), and dried in vacuo. [Cr(Phen)$_2$(4,4'-d^{t}bpy)](OTf)$_3$ was isolated as a yellow powder (0.46 g, 42 %). IR: $\nu_{C=N}$ 1619 cm^{-1}. ES$^+$MS (CH$_3$CN): *m/z* 978.0 ([**5** – OTf]$^+$), 226.9 ([**5** – 3OTf]$^{3+}$). Anal. Calcd. for $C_{45}H_{40}N_6CrF_9O_9S_3$: C, 47.91; H, 3.58; N, 7.45. Found: C, 47.71; H, 3.72; N, 7.62 %.

Synthesis of $[Cr(Phen)_2(4,4'\text{-dmcbpy})](OTf)_3$ (6)

$[Cr(Phen)_2(CF_3SO_3)_2](CF_3SO_3)$ (0.68 g, 0.79 mmol) and 4,4'-dmcbpy (322 mg, 1.18 mmol) were suspended in CH_2Cl_2 (60 ml). The reaction mixture was heated to reflux. Over 10 days a bright yellow precipitate was formed which was separated by filtration, washed with CH_2Cl_2 (2 x 15 ml), and dried in vacuo. $[Cr(Phen)_2(4,4'\text{-dmcbpy})](OTf)_3$ was isolated as a bright yellow powder (0.22 g, 25 %). IR: $\nu_{C=N}$ 1624, $\nu_{C=O}$ 1731 cm^{-1}. ES^+MS (CH_3CN): *m/z* 228.30 ([**6** – $3OTf]^{3+}$). Anal. Calcd. for $C_{41}H_{28}N_6CrF_9O_{13}S_3$: C, 43.51; H, 2.49; N, 7.42. Found: C, 43.78; H, 2.33; N, 7.24 %.

Synthesis of $[Cr(Phen)_2(5,5'\text{-DMB})](OTf)_3$ (7)

$[Cr(Phen)_2(CF_3SO_3)_2](CF_3SO_3)$ (0.74 g, 0.86 mmol) and 5,5'-Me_2bpy (256 mg, 1.39 mmol) were suspended in CH_2Cl_2 (60 ml). The reaction mixture was heated to reflux for 36 h and a yellow precipitate was formed. This was separated by filtration, washed with CH_2Cl_2 (2 x 15 ml), and dried in vacuo. $[Cr(Phen)_2(5,5'\text{-DMB})](OTf)_3$ was isolated as a yellow powder (0.60 g, 58 %). IR: $\nu_{C=N}$ 1608 cm^{-1}. ES^+MS (CH_3CN): *m/z* 894.0 ([**7** – $OTf]^+$), 198.8 ([**7** – $3OTf]^{3+}$). Anal. Calcd. for $C_{39}H_{28}N_6CrF_9O_9S_3$: C, 44.87; H, 2.71; N, 8.05. Found: C, 44.52; H, 2.65; N, 7.93 %.

Synthesis of $[Cr(Phen)_2(5\text{-Me-Phen}](OTf)_3$ (8)

$[Cr(Phen)_2(CF_3SO_3)_2](CF_3SO_3)$ (0.79 g, 0.92 mmol) and 5-Me-Phen (247 mg, 1.27 mmol) were suspended in CH_2Cl_2 (80 ml). The reaction mixture was heated to reflux for 40 h and a yellow precipitate was formed. This was separated by filtration, washed with CH_2Cl_2 (2 × 15 ml), and dried in vacuo. $[Cr(Phen)_2(5\text{-Me-Phen})](OTf)_3$ was isolated as a yellow powder (0.49 g, 44 %). IR: $\nu_{C=N}$ 1605 cm^{-1}. ES^+MS (CH_3CN): *m/z* 904.0 ([8 – $OTf]^+$), 202.1 ([8 – $3OTf]^{3+}$). Anal. Calcd. for $C_{40}H_{26}N_6CrF_9O_9S_3$: C, 45.58; H, 2.49; N, 7.98. Found: C, 45.44; H, 2.29; N, 7.83 %.

Synthesis of $[Cr(Phen)_2(4,7\text{-DMP})](OTf)_3$ (9)

$[Cr(Phen)_2(CF_3SO_3)_2](CF_3SO_3)$ (0.75 g, 0.87 mmol) and 4,7-DMP (272 mg, 1.31 mmol) were suspended in CH_2Cl_2 (60 ml). The reaction mixture was heated to reflux for 7 days, and a yellow precipitate was formed which was separated by filtration, washed with CH_2Cl_2 (2 × 15 ml), and dried in vacuo. $[Cr(Phen)_2(4,7\text{-DMP})](OTf)_3$ was isolated as a yellow powder (0.21 g, 20 %). IR: $\nu_{C=N}$ 1610 cm^{-1}. ES^+MS (CH_3CN): *m/z* 918.0 ($[\mathbf{9} - OTf]^+$), 206.8 ($[\mathbf{9} - 3OTf]^{3+}$). Anal. Calcd. for $C_{41}H_{28}N_6CrF_9O_9S_3$: C, 46.11; H, 2.65; N, 7.87. Found: C, 45.83; H, 2.47; N, 7.64 %.

REFERENCES

(1) McDaniel, A. M.; Tseng, H.-W.; Damrauer, N. H.; Shores, M. P. Synthesis and solution phase characterization of strongly photooxidizing heteroleptic Cr(III) tris-dipyridyl complexes. *Inorg. Chem.* **2010**, *49* (17), 7981-7991.
